# Segmentation gene expression and function in *Vanessa cardui*, an emerging model for Lepidoptera

**DOI:** 10.1242/dev.205835

**Published:** 2026-10-12

**Authors:** Ximena Gutiérrez Ramos, Katie Reding, Leslie Pick

**Affiliations:** Department of Entomology, University of Maryland, College Park, MD 20742, USA

**Keywords:** *Vanessa cardui*, Pair-rule genes, Segmentation, *Blimp1*

## Abstract

Although all insects are segmented, the genes that control this process vary across species. Many of the pair-rule (PR) genes that direct segment formation in *Drosophila* are similarly utilized in other holometabolous insects, but more distantly related species use different genes for PR patterning. Previously, we showed that Lepidoptera lack a highly conserved PR gene, *paired.* Here, we used the painted lady butterfly *Vanessa cardui* as a lepidopteran model to explore the expression and function of PR genes in this large clade of moths and butterflies. Orthologs of four *Drosophila* PR genes are expressed in PR-like stripes and at least one displays PR-like function. Neither of the two genes that have PR function in Hemiptera but not in *Drosophila* has a PR role in *Vanessa.* Rather, the hemipteran PR-gene *Blimp1* functions in a novel fashion in abdominal segmentation in *Vanessa*. Thus, while butterflies appear to share PR-patterning mechanisms with other insects, they utilize only a subset of the *Drosophila* PR-gene orthologs and have not taken on hemipteran PR orthologs for this process. How *Vanessa* patterns segments with a more limited set of PR-gene orthologs remains to be determined.

## Teaser

**Highlighted Article:** Mechanisms controlling embryonic development are underexplored in Lepidoptera. Here we show that only some of the known segmentation genes contribute to body patterning in the model species, painted lady butterfly.

## INTRODUCTION

The organization of the basic body plan into repeated segments along the anterior-posterior (AP)-axis is shared across bilaterian animals (reviewed by Blair, 2008; Diaz-Cuadros et al., 2021). This process has been studied in diverse taxa but, arguably, is best understood in the insect model species *Drosophila melanogaster* (Wieschaus and Nüsslein-Volhard, 2016). Sets of regulatory genes act in a well-studied hierarchy to generate and then diversify *Drosophila* body segments (Barresi and Gilbert, 2023). Mutations in the pair-rule (PR) genes result in loss of alternate segment-wide units across the AP axis, indicative of a double-segment-wide prepattern early in development (Nüsslein-Volhard and Wieschaus, 1980). In *Drosophila*, most PR genes are expressed in seven-stripe patterns in early embryos, in the primordia of the alternate segmental regions they specify (Barresi and Gilbert, 2023). However, broadly expressed genes can still function as PR genes; for example, *fushi tarazu factor 1* (*ftz-f1*) is expressed ubiquitously but its PR function is restricted by the striped expression of its obligate partner Fushi Tarazu (Ftz) (Yu et al., 1997; Guichet et al., 1997; Yussa et al., 2001).

Although this segmentation gene network was worked out in *Drosophila*, it is reasonable to expect that segmentation would be regulated by the same genes in other insects, given that all insects are segmented. However, there is significant variation in the timing of segment establishment across insects, which may suggest corresponding variability in genetic regulation. Segments in *Drosophila* are established more-or-less simultaneously at the blastoderm stage (simultaneous segmentation). Many other insect species establish only the anterior-most segments in early blastoderm and add remaining segments one at a time as the germband elongates (sequential segmentation; reviewed by Davis and Patel, 2002; Liu and Kaufman, 2005), leading to the question of whether segmentation in sequentially segmenting insects is regulated by the same gene network as in simultaneous-segmenting insects.

This question has been most thoroughly addressed in beetles. In *Tribolium*, orthologs of most *Drosophila* PR genes are expressed in striped patterns (Sommer and Tautz, 1993; Brown et al., 1994; Patel et al., 1994; Brown and Denell, 1996; Maderspacher et al., 1998; Choe et al., 2006, 2017; Choe and Brown, 2007; Aranda et al., 2008; Janssen et al., 2010; Heffer et al., 2013; Clark and Peel, 2018). RNA interference (RNAi) knockdown of *Tribolium even-skipped* (*eve*), *runt* (*run*) or *odd-skipped* (*odd*) resulted in truncated embryos that displayed little more than a head, suggesting that these genes are required for germband elongation (Choe et al., 2006). Similar head-only phenotypes were obtained in a second beetle species, *Dermestes maculatus* (Xiang et al., 2017). However, weaker RNAi knockdown revealed segmental fusions and deletions in PR registers for these genes (Xiang et al., 2017). Thus, in beetles, strong knockdown of three PR genes resulted in inhibition of germband elongation and produced a truncated embryo, while weaker knockdowns that allowed germband elongation to proceed caused *Drosophila*-like PR defects. In *Tribolium*, only two PR mutants were isolated in a mutant screen, corresponding to *paired* (*prd*) and *sloppy paired* (*slp*), the two genes with exclusively PR-like defects after RNAi knockdown in *Dermestes* (Maderspacher et al., 1998; Choe and Brown, 2007; Xiang et al., 2015, 2017). Variation in expression and/or function in either one or both beetle species was found for other PR orthologs (Stuart et al., 1991; Aranda et al., 2008; Heffer et al., 2013; Choe et al., 2017; Xiang et al., 2017; Clark and Peel, 2018). Collectively, these results suggest that, while much of the regulatory network coordinating segmentation in *Drosophila* is conserved in sequentially segmenting beetles, it is not precisely recapitulated.

More limited studies have been carried out in other holometabolous insects, including the wasp *Nasonia vitrippennis* and the honeybee *Apis mellifera*, in which several PR orthologs have taken on additional roles (Binner and Sander, 1997; Osborne and Dearden, 2005; Dearden et al., 2006; Keller et al., 2010; Wilson et al., 2010; Wilson and Dearden, 2012; Rosenberg et al., 2014; Taylor and Dearden, 2022; see also Discussion). In the mosquito *Anopheles stephensi*, the *prd* gene has been lost, but the *Pax3/7* family member *gooseberry* (*gsb*) has taken on PR-like expression and functionally replaced *prd* (Cheatle Jarvela et al., 2020), a clear example of how gene loss can be permitted after functional redundancy is established*.* In previous phylogenetic analysis of the Pax genes, we showed that *prd* is also absent from lepidopteran genomes (Cheatle Jarvela et al., 2020; Gutiérrez Ramos and Pick, 2025). However, *gsb* was found in Lepidoptera. In fact, *gsb* appears to be conserved across all arthropod species, and is thus more broadly conserved than *prd* (Gutiérrez Ramos and Pick, 2025).

Less is known about segmentation in the order Lepidoptera, which includes butterflies and moths. Lepidopterans appear to form most of their segments sequentially from a posterior segment addition zone (SAZ) (Kraft and Jäckle, 1994; and see below). Some *Drosophila* PR-gene orthologs have been studied in the moths *Bombyx mori* and *Manduca sexta*, revealing conservation of PR expression and function (see Discussion), but we have not found reports of PR gene expression in early embryos of any butterfly.

Here, we used techniques that we previously established in the painted lady butterfly, *Vanessa cardui* (*Vcar*) (Gutiérrez Ramos and Pick, 2025), to examine PR gene expression and function in lepidopterans. We found that *Vanessa gsb* is expressed and functions in segmental stripes, similar to *Drosophila* (Nüsslein-Volhard and Wieschaus, 1980; Bopp et al., 1986; Baumgartner et al., 1987). Orthologs of four PR genes have PR-like expression in *Vanessa* and RNAi knockdown of *Vcar*-*eve*, and possibly *Vcar*-*run*, revealed PR-like function. We also examined *Vanessa E75* and *Blimp1*, genes that function as PR genes in the hemimetabolous insect *Oncopeltus fasciatus* (Erezyilmaz et al., 2009; Reding et al., 2024). RNAi knockdown of *Vcar-Blimp1* revealed a novel phenotype, with deletion of two abdominal segments. Overall, *Vanessa* appears to utilize a subset of the *Drosophila* PR genes, but none of the *Oncopeltus* orthologs, for PR patterning. How *Vanessa*, and possibly other Lepidoptera, pattern segments with a more limited or as-yet-undiscovered set of genes remains to be determined.

## RESULTS

### *V. cardui* early embryo development

We examined the timing and stages of *Vanessa* development from egg laying to 16 h after egg laying (AEL). *Vanessa* eggs are fertilized as they are laid (Fig. 1Ai). Nuclear divisions start in the most anterior part of the egg (Fig. 1Ai, arrowhead) and later distribute across the embryo. The first nuclear divisions were observed from 0 to 4 h AEL (Fig. 1Aii,Ci) and continued until 6 h AEL (Fig. 1Aiii,Aiv). The blastoderm formed at ∼8 h AEL (Fig. 1Av,Bi), characterized by a broad sheet of cells along the circumference of the egg (Fig. 1Bi,Cii). Extra-embryonic tissue is distinguishable by large nuclei in anterior and posterior poles (Fig. 1Bi, arrowheads). Next, the blastoderm compressed laterally, initiating germ layer formation within the germ primordium; the anterior margins of the blastoderm invaginated to form the head lobes. At this stage, the shape of the primordium resembled a saddle, with the SAZ at the posterior end (Fig. 1Bii,Ciii, S). When the germband started to elongate, the protocephalon (Fig. 1Biii,Biv,Civ, asterisks) and the primitive midline groove were distinguishable. The germband continued to elongate, with segments added sequentially until ∼16 h AEL, when the gnathal segments, the head lobes and the midline were evident (Fig. 1Bv,Cv,Cvi).

**Fig. 1.**
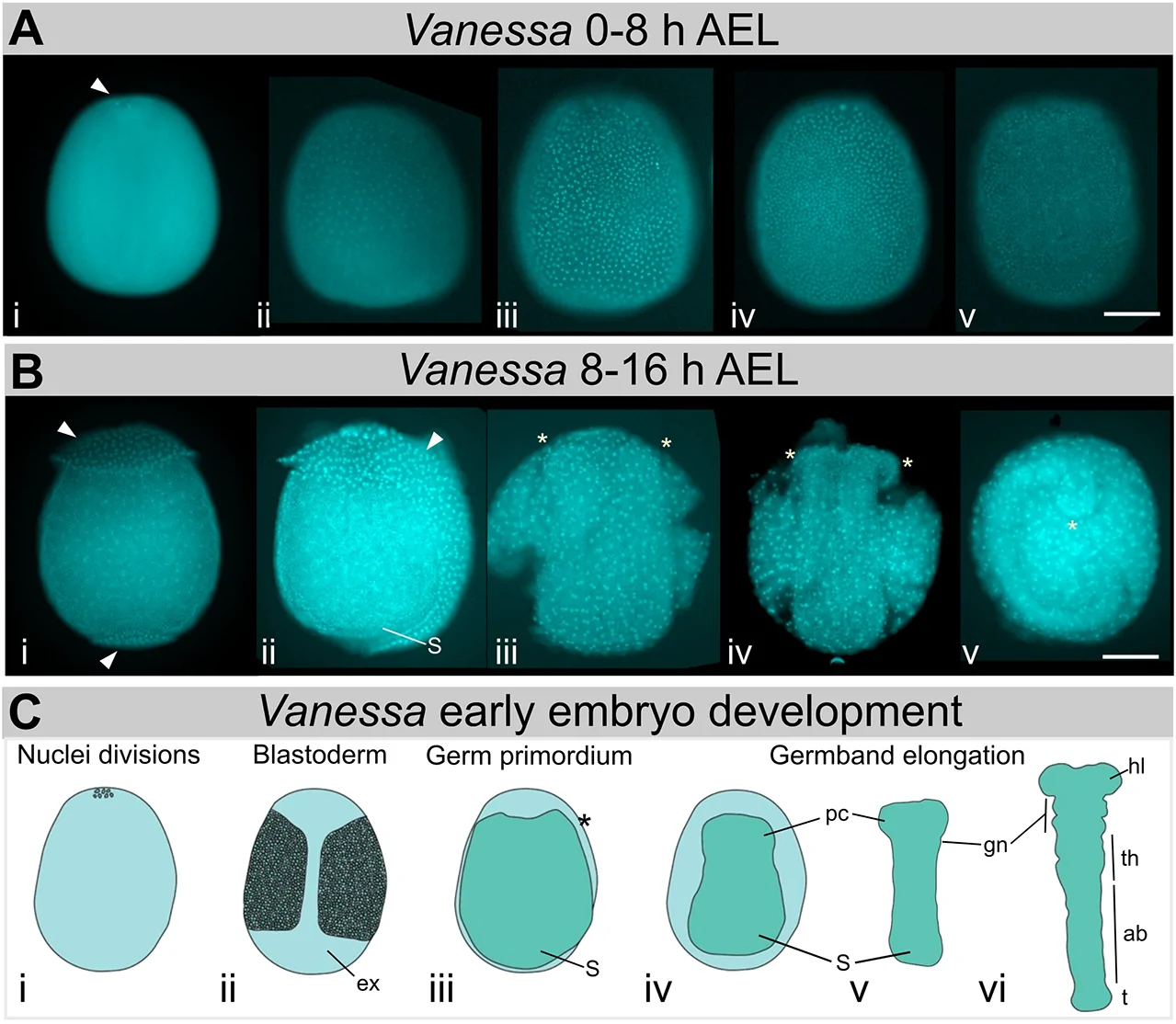
***Vanessa* early embryo development.** (A,B) Hoechst nucleic acid staining of embryos at successive stages of development. (A) 0 to 8 h AEL. Arrowhead indicates first nuclear divisions (i). (B) 8 to 16 h AEL. Arrowheads indicate extra-embryonic tissue (i,ii). Asterisks indicate protocephalon (iii,iv). (C) Schematic of the main developmental changes across the first 16 h of *Vanessa* embryo development. Asterisk indicates protocephalon (iii). ab, abdominal segments; ex, extra-embryonic tissue; gn, gnathal segments; hl, head lobes; pc, protocephalon; S, SAZ; t, telson; th, thoracic segments. Images orientated anterior top, posterior bottom. Scale bars: 200 µm.

This early embryonic developmental progression is like that seen for other Lepidopterans, such as *Bombyx* (Krause and Krause, 1964; Miya, 1985; Nagy et al., 1994), *Manduca* (Dorn et al., 1987; Broadie et al., 1991; Kraft and Jäckle, 1994) and *Bicyclus* (Holzem et al., 2019). However, we were unable to distinguish germinal disc formation like that localized in the anterior of *Bicyclus* embryos at 5 h AEL (Holzem et al., 2019). Our experiments defined the timing of blastoderm formation as ∼8 h AEL, likely the earliest stage at which PR gene expression would be expected.

### *Vanessa-gsb* is a segment polarity gene

We previously showed that *Vcar-gsb* is expressed in stripes with single-segment periodicity during germband elongation (Gutiérrez Ramos and Pick, 2025). As *gsb* has been shown to have PR function in a mosquito (Cheatle Jarvela et al., 2020), we investigated whether *Vcar-gsb* has also taken over PR function from *prd*, or has segment polarity function as in *Drosophila*, or some other function. We expanded our sampling of developmental stages to characterize *Vcar-gsb* expression from 8 to 16 h AEL. The earliest pattern detected was two anterior stripes (Fig. 2Ai, arrowheads). With the separation of the protocephalon (Fig. 2Aii, asterisks), the anterior-most stripe split medially and additional stripes were observed in the primordium of each segment (Fig. 2Aii). No stripes were observed in the SAZ (Fig. 2Aii,Aiii). As the germband elongated, each stripe appeared to split medially (Fig. 2Aiv,Av), as previously observed (Gutiérrez Ramos and Pick, 2025). Thus, *Vcar-gsb* is expressed in the blastoderm and through germband elongation with single-segment periodicity.

**Fig. 2.**
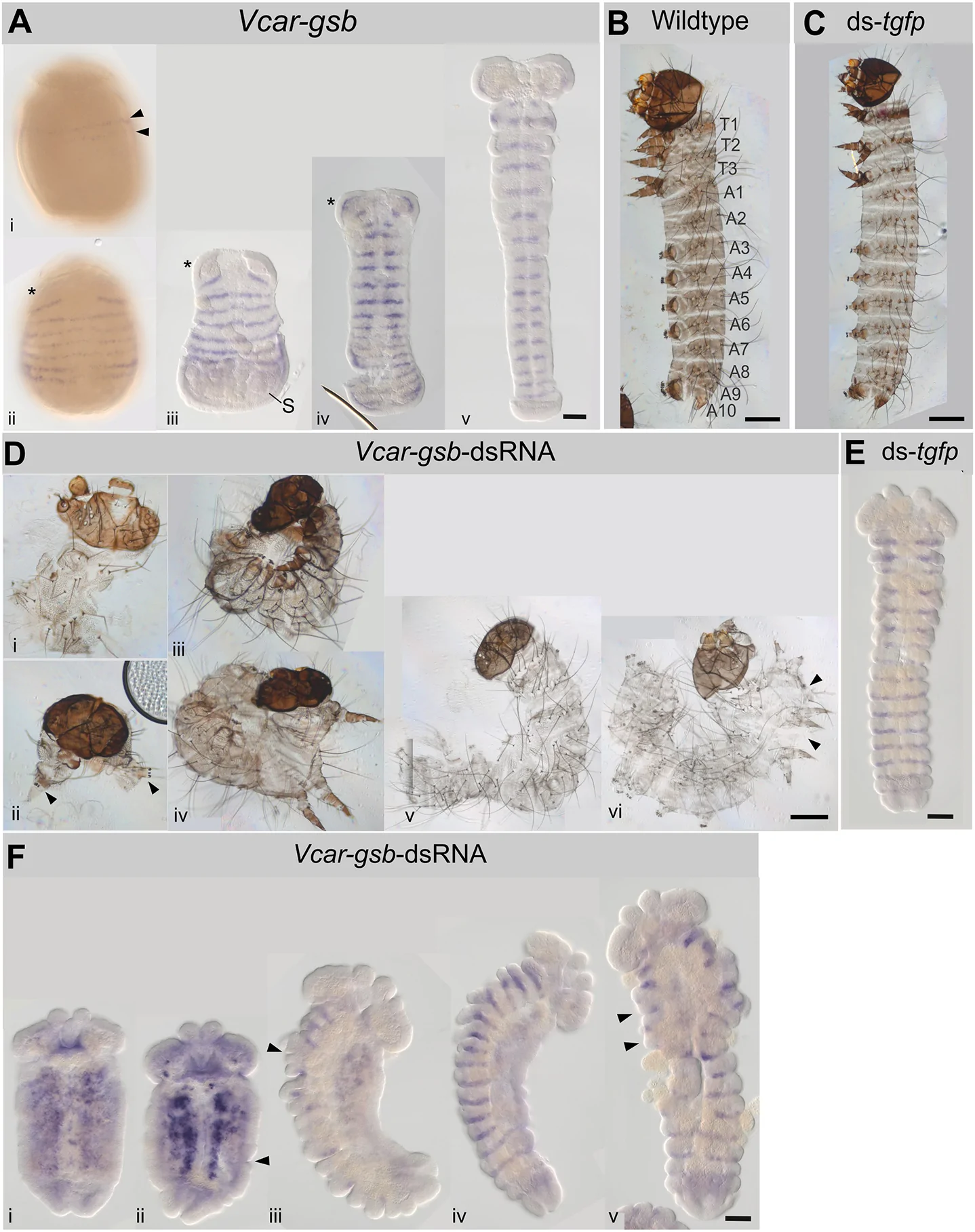
***Vcar-gsb* displays striped expression and segment polarity-like function.** (A) Colorimetric *in situ* hybridization with *Vcar-gsb* probe at successive time points between 8 and 16 h AEL. (i) Anterior blastoderm stripes (arrowheads). (ii) Multiple stripes in germ primordium, with the two most-anterior stripes separating (asterisk). (iii) Early elongating germbands: segmental stripes; stripe in head lobes separated medially (asterisk); no expression observed in the SAZ (S). (iv,v) Stripes in every segment; faint expression in head lobes (asterisk). (B) Cuticle preparation from *Vanessa* wild-type larva: head, three thoracic segments (T1-T3), ten abdominal segments (A1-A10). A3-A6 and A10 each bear a pair of prolegs. (C) ds-*tgfp* eRNAi embryo displaying a wild type-like phenotype. (D) *Vcar-gsb*-dsRNA eRNAi embryos: (i) head phenotype, (ii) head with one set of prolegs (arrowhead), (iii,iv) torus, (v,vi) partial fused segments (arrowheads). (E,F) *Vcar-en in situ* hybridization in *tgfp*-dsRNA eRNAi (E) and *Vcar-gsb*-dsRNA eRNAi (F) embryos. Arrowheads indicate fused segments. Images orientated anterior, top; posterior, bottom. Scale bars: 200 µm (B-D); 100 µm (A,E,F).

We carried out embryonic RNAi (eRNAi) knockdown of *Vcar-gsb* using two non-overlapping double-stranded RNAs (dsRNA), both targeting regions downstream of the homeobox (dsRNA1 and dsRNA2; Fig. S1A). Embryos were injected with *Vcar-gsb* dsRNA1, *Vcar-gsb* dsRNA2 or *tgfp* dsRNA (ds-*tgfp*) as a negative control, and allowed to develop until just before wild-type embryos would be expected to hatch (‘prehatchlings’). *Vanessa* wild-type prehatchlings have a head, three thoracic segments (T1-T3) and ten abdominal segments (A1-A10), with A3-A6 and A10 each possessing a pair of prolegs (Fig. 2B, wild type). After control ds-*tgfp* injection, the expected segments were observed in 86% of injected embryos (*n*=452/523; Fig. 2C). No development was observed for 13% (*n*=66/523) of injected embryos and 1% showed delayed development (*n*=5/523). Knockdown of *Vcar-gsb* resulted in four key phenotypes (Fig. 2D), observed in 85% (*n*=384/450) of dsRNA1-injected embryos and 81% (*n*=468/581) of dsRNA2-injected embryos (see details in Table S1): (1) The most severe phenotype, seen in ∼25% of prehatchlings, termed ‘head-like’ displayed the head capsule with stemmata, frons and antennae similar to wild type while mandibles, maxillae and labrum appeared distinct from wild type (Fig. 2Di); (2) Less severe truncations, seen in ∼20% of prehatchlings, consisted of the head, undefined tissue and one set of prolegs (Fig. 2Dii, arrowheads); (3) ∼30% of prehatchlings displayed a torus-like shape, with thoracic and abdominal segments fused on just one side of the embryo, presumably causing the observed curvature (Fig. 2Diii, ventral view; Fig. 2Div, dorsal view of a different embryo); (4) Other prehatchlings had partial segmental fusions – in some cases A1-A4 were fused on one side of the embryo (Fig. 2Dv), in others thoracic segments were fused on one side of the embryo (Fig. 2Dvi), suggesting intermediate forms of the torus phenotype. Overall, segment formation defects were observed after *gsb* knockdown in more than 80% of embryos examined but no PR-like defects were observed, although we cannot rule out the possibility that the head-only phenotypes reflect a PR-like role.

Next, we examined whether high concentrations of the dsRNAs masked potential early PR function. We performed injections of *Vcar-gsb*-dsRNA1 at lower concentrations. The most severe phenotypes previously observed after knockdown with *Vcar-gsb*-dsRNA1 (head-like, head with one set of prolegs, and torus) were absent when 0.15 µM or 0.015 µM dsRNA was injected. Embryos injected with *Vcar-gsb*-dsRNA1 at 0.15 µM showed partial fused segments (41%, *n*=55/133), wild type-like phenotypes (35%, *n*=47/133), developmental delay (19%, *n*=25/133) or no development (5%, *n*=6/133). At 0.015 µM, the frequency of partially fused segments decreased to 23% (*n*=33/142) and the frequency of wild type-like phenotypes increased to 64% (*n*=91/142) (Table S3). However, we did not observe a PR-like loss of alternate segments, although the specific identities of segments present in larvae with torus or with partial fusion phenotypes could not be definitively determined.

We also performed *in situ* hybridizations in *Vcar-gsb* dsRNA-injected embryos to visualize expression of *Vcar engrailed* (*Vcar-en*), a conserved segment polarity gene (Wieschaus and Nüsslein-Volhard, 2016). In ds-*tgfp* control embryos, *Vcar-en* stripes were present in every segment of elongated germbands (Fig. 2E), as expected. In *Vcar-gsb*-dsRNA1 embryos, we observed a range of abnormalities, including embryos with head lobes and telson, but no defined segments (Fig. 2Fi,Fii) with *Vcar-en* expression concentrated into partially fused rows along each side of the midline, possibly due to fusion of multiple stripes along the AP axis and/or failure of germband elongation. Some embryos displayed a well-defined head, telson and lateral segments, with some fused segments (Fig. 2Fiii, arrowhead), with *Vcar-en* stripes visible in several segments. Other embryos completely lacked segmentation and *en* expression in one lateral half of the body, while displaying wild type-like *en* stripes in every segment in the other half (Fig. 2Fiv). Finally, some embryos displayed partially fused segments, with *Vcar-en* stripes in only some segments (Fig. 2Fv). We did not observe a PR-like register to the segmental fusions in these embryos. Together, these findings suggest that *Vcar-gsb* is required for the formation of all the segments, similar to its role as a segment polarity gene in *Drosophila*.

### Some *Vanessa* PR gene orthologs are expressed in PR-like stripes

Given the lack of an obvious PR role for *Vcar-gsb*, and the absence of *prd* from the genome, we examined whether *Vanessa* use PR patterning per se and, if so, whether orthologs of other *Drosophila* PR genes drive this process. We identified *Vanessa* orthologs of *ftz*, *ftz-f1*, *eve*, *sloppy paired 1* (*slp1*), *sloppy paired 2* (*slp2*), *run*, *odd*, *hairy* (*h*) and *odd-paired* (*opa*) (Table S6, Fig. S2) and performed *in situ* hybridization for each in 8-16 h AEL embryos.

*Vcar-ftz* expression was observed as a wide band in the posterior part of the germ primordium (Fig. 3Ai, arrowhead), followed by one anterior wide band, one posterior band, and expression in the SAZ (Fig. 3Aii). Once the germband elongated, expression continued in the SAZ and midline of the germband (Fig. 3Aiii). Later, expression appeared in the midline as spots in every segment, presumably in the developing nervous system (Fig. 3Aiv,Av). *Vcar-ftz-f1* expression was not detected in the blastoderm or germ primordium (Fig. 3Bi,Bii) and later appeared to be faintly, ubiquitously expressed (Fig. 3Biii,Bv).

**Fig. 3.**
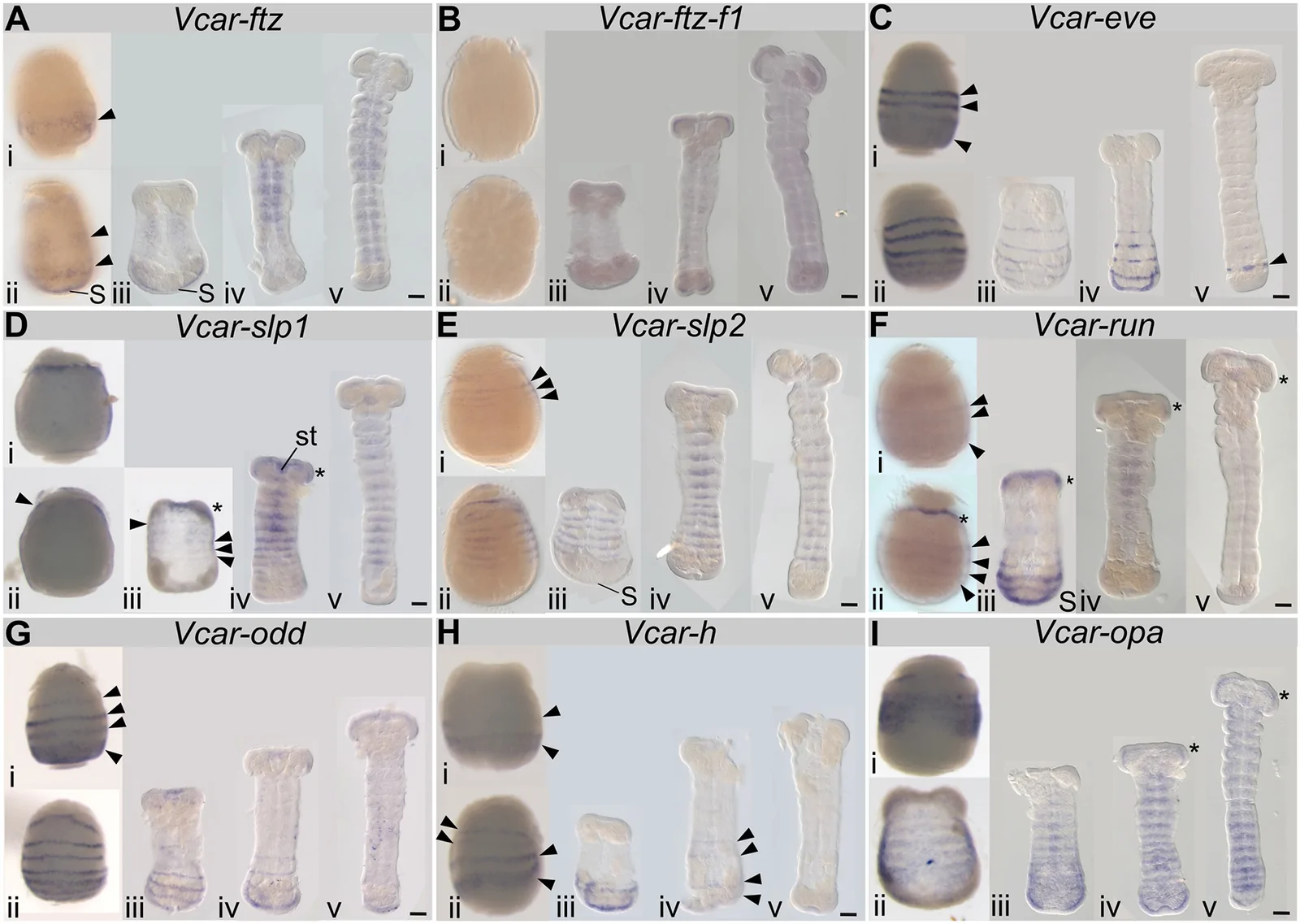
**Expression patterns of *Vanessa* PR gene orthologs.** Colorimetric *in situ* hybridization with *Vcar* PR gene probes, as indicated at (i,ii) blastoderm stage, germ primordia, and (iii-v) successive stages of germband development. (A) *Vcar-ftz*: (i) posterior band (arrowhead); (ii) two widely spaced bands (arrowheads), SAZ (S) expression; (iii-v) expression in midline, likely the developing nervous system. (B) *Vcar-ftz-f1*: (i,ii) not detected; (iii-v) faint ubiquitous expression in elongated germbands. (C) *Vcar-eve*: (i) two widely spaced anterior stripes and broad posterior band (arrowheads); (ii) five stripes along the germband; (iii) four stripes in the posterior of the embryo; (iv) expression at the posterior SAZ in fully elongated germbands; (v) one stripe anterior to the SAZ (arrowhead). (D) *Vcar-slp1*: not detected in blastoderm and protocephalon (arrowhead) expression in germ primodia (i,ii). Stripes along the elongating germband (iii, arrowheads) are later clear in every segment, head lobes (asterisk) and stomodeum (st) (iv). (v) Striped expression maintained in thoracic and abdominal segments. (E) *Vcar-slp2*: (i) anterior stripes (arrowheads); (ii) additional stripes present; (iii,iv) segmental stripes with no expression observed in SAZ; (v) stripes centrally localized. (F) *Vcar-run*: (i) two widely spaced anterior stripes and a broad posterior stripe (arrowheads); (ii) additional stripes in germ primordium (arrowheads); (iii) expression in head lobes (asterisk) and posterior stripes, likely SAZ (S); (iv,v) expression in head lobes (asterisks), midline paired dots in gnathal and thoracic segments, stripes in SAZ. (G) *Vcar-odd*: (i) three widely spaced anterior stripes and posterior broad band (arrowheads); (ii) widely spaced stripes along germ primordium; (iii,iv) striped expression continuing in the posterior germband and (v) fading at the end of the 16 h AEL. (H) *Vcar-h*: (i,ii) two or three widely spaced stripes in blastoderm (arrowheads); (iii) stripes in posterior elongating germband and SAZ; (iv) stripes still detectable (arrowheads); (v) no expression observed by 16 h AEL. (I) *Vcar-opa*: (i) broad anterior band; (ii) four stripes along the germ primordium; (iii) segmental stripes, expression in SAZ and along the midline; (iv) expression in head lobes (asterisk), segmental stripes and SAZ; (v) expression in head lobes (asterisk), segmental stripes in abdominal segments. Images orientated anterior top, posterior bottom. Scale bars: 100 µm.

*Vcar-eve* expression was first detected in the blastoderm as two anterior stripes and a wide band in the posterior (Fig. 3Ci, arrowheads). Later, this posterior band appeared to split into three separate stripes (Fig. 3Cii), resulting in five bands across the germband primordia. *Vcar-eve* stripes in the germband were widely spaced (Fig. 3Ciii,Civ), especially compared to *gsb* expression in embryos of similar age (compare Fig. 3Civ to Fig. 2Aiv). As the germband elongated, stripes were seen only in the most posterior part of the germband (Fig. 3Civ). In older germbands, *Vcar-eve* expression was observed in a single stripe close to the telson (Fig. 3Cv, arrowhead).

*Vcar-slp1* expression was not detected in the blastoderm (Fig. 3Di). In the germ primordium, anterior expression was observed in the protocephalon (Fig. 3Dii, arrowhead), head lobes (asterisk), stomodeum and in stripes along the germband (arrowheads) (Fig. 3Diii,Div). Stripes were present as the germband elongated, with apparent segmental register (Fig. 3Div,Dv). *Vcar-slp2* striped expression first appeared in the anterior part of the blastoderm (Fig. 3Ei, arrowheads), and later stripes arose along the germ primordium with no expression detected in the SAZ (Fig. 3Eii,Eiii). During germband elongation, *Vcar-slp2* was expressed in centrally localized stripes marking each segment (Fig. 3Eiv,Ev).

*Vcar-run* expression was observed in the blastoderm as two anterior stripes and a wide posterior band (Fig. 3Fi, arrowheads). In the germ primordium, widely spaced stripes of expression were observed (Fig. 3Fii, arrowheads), similar to *Vcar-eve* in a similarly staged embryo (compare Fig. 3Fii to Cii). During early germband elongation, expression was observed in the head lobes, in stripes in the posterior of the germband and in the SAZ (Fig. 3Fiii). Expression continued in the head lobes, with segmental, centrally located stripes in the gnathal and thoracic segments (Fig. 3Fiv). Later, faint expression continued in the head lobes and stomodeum (Fig. 3Fv, asterisk).

*Vcar-odd* expression was first observed in the blastoderm as three stripes and a wide posterior band (Fig. 3Gi, arrowheads), similar to *Vcar-eve*. This wide band later resolved into several stripes (Fig. 3Gii). When the germband started to elongate, striped expression was only observed in the posterior of the embryos (Fig. 3Giii,Giv). Expression was not detected in later germbands (Fig. 3Gv).

*Vcar-h* blastoderm expression was first observed in stripes in the posterior half of the embryo (Fig. 3Hi, arrowheads). Later, four widely spaced stripes of varying intensity were observed (Fig. 3Hii). At the beginning of germband elongation, *Vcar-h* expression was observed in the SAZ (Fig. 3Hiii). *Vcar-h* stripes faded in the elongated germband (Fig. 3Hiv, arrowheads) and were undetectable in older embryos (Fig. 3Hv).

*Vcar-opa* expression appeared in the blastoderm as an anterior, wide band that resolved into stripes along the germ primordium (Fig. 3Ii,Iii). In the elongated germband, striped expression was observed, in addition to expression along the midline and in the SAZ (Fig. 3Iiii). Later, expression was seen in the head lobes, in segmental stripes and in the SAZ (Fig. 3Iiv). At the end of 16 h AEL, expression in the head lobes, and gnathal and thoracic segments appeared weaker, while segmental striped expression in the abdominal segments continued (Fig. 3Iv).

Based on these expression patterns, we can classify *Vanessa* PR genes in groups: (1) widely spaced stripes suggestive of PR-like expression for *Vcar-eve*, *Vcar-run*, *Vcar-odd* and *Vcar-h*; (2) segmental stripes for *Vcar-slp2* and *Vcar-opa*, with segmental stripes appearing later for *Vcar-slp1*; and (3) *Vcar-ftz*, which was difficult to classify with widely spaced stripes in the blastoderm and presumptive central nervous system expression. *Vcar-ftz-f1* expression was very faint in embryos and we were not able to isolate it from early embryonic cDNA.

### *Vanessa eve* RNAi suggests classical PR function

We next selected two genes that have PR-like expression, *Vcar-eve* and *Vcar-run*, and one with segmental expression, *Vcar-slp*, to assess function. For knockdown of *Vcar-eve*, we designed two non-overlapping dsRNAs, with dsRNA1 targeting most of the homeobox and dsRNA2 targeting the end of homeobox to the end of the transcript (Fig. S1D). *Vanessa* embryos injected with ds-*tgfp* as negative control showed wild type-like phenotypes in 86% (*n*=452/523) of embryos (Fig. 4A, Table S1). We observed three distinct segmentation phenotypes following *Vcar-eve*-dsRNA knockdown, in total comprising 55% (*n*=299/540) of dsRNA1-injected embryos and 59% (*n*=267/450) of dsRNA2-injected embryos (Table S1): (1) The PR-like loss of the even-numbered abdominal segments (Fig. 4B). Segments were identified by the presence of only one pair of legs (identifying T2) and the presence of only two pairs of prolegs, identifying segments A3 and A5 (31%, *n*=167/540, with dsRNA1; 39%, *n*=175/450, with dsRNA2). (2) Head-like, similar to those seen after knockdown of *Vcar-gsb* (1%, *n*=4/540, with dsRNA1; 4%, *n*=20/450, with dsRNA2) (Fig. S3Ai). (3) Partial fusions, including loss of two thoracic segments, presumably T1 and T3, and one abdominal segment with three sets of prolegs remaining (24%, *n*=128/540, with dsRNA1; 16%, *n*=72/450, with dsRNA2) (Fig. S3Aii, arrowheads). Others displayed fusion of thoracic segments, leaving just three legs (Fig. S3Aiii,Aiv, asterisks), and all abdominal segments (Fig. S3Aiii,Aiv, arrowheads). Partial fusions suggest an intermediate form of the PR-like phenotype. The range of phenotypes observed is typical for RNAi knockdown, which generates the equivalent of an allelic series due to differences in dose or timing of dsRNA action in different embryos. For beetles, knockdown of *eve* generated a similar range of defects with strong knockdown resulting in head-only phenotypes and weak knockdown producing partial segmental fusions with PR register (Choe et al., 2006; Xiang et al., 2017). The truncations seen for *eve* and other PR genes in sequentially segmenting species likely reflect a role not required in simultaneously segmenting species such as *Drosophila.* In sum, these results suggest that *Vcar-eve* has PR function in *Vanessa*.

**Fig. 4.**
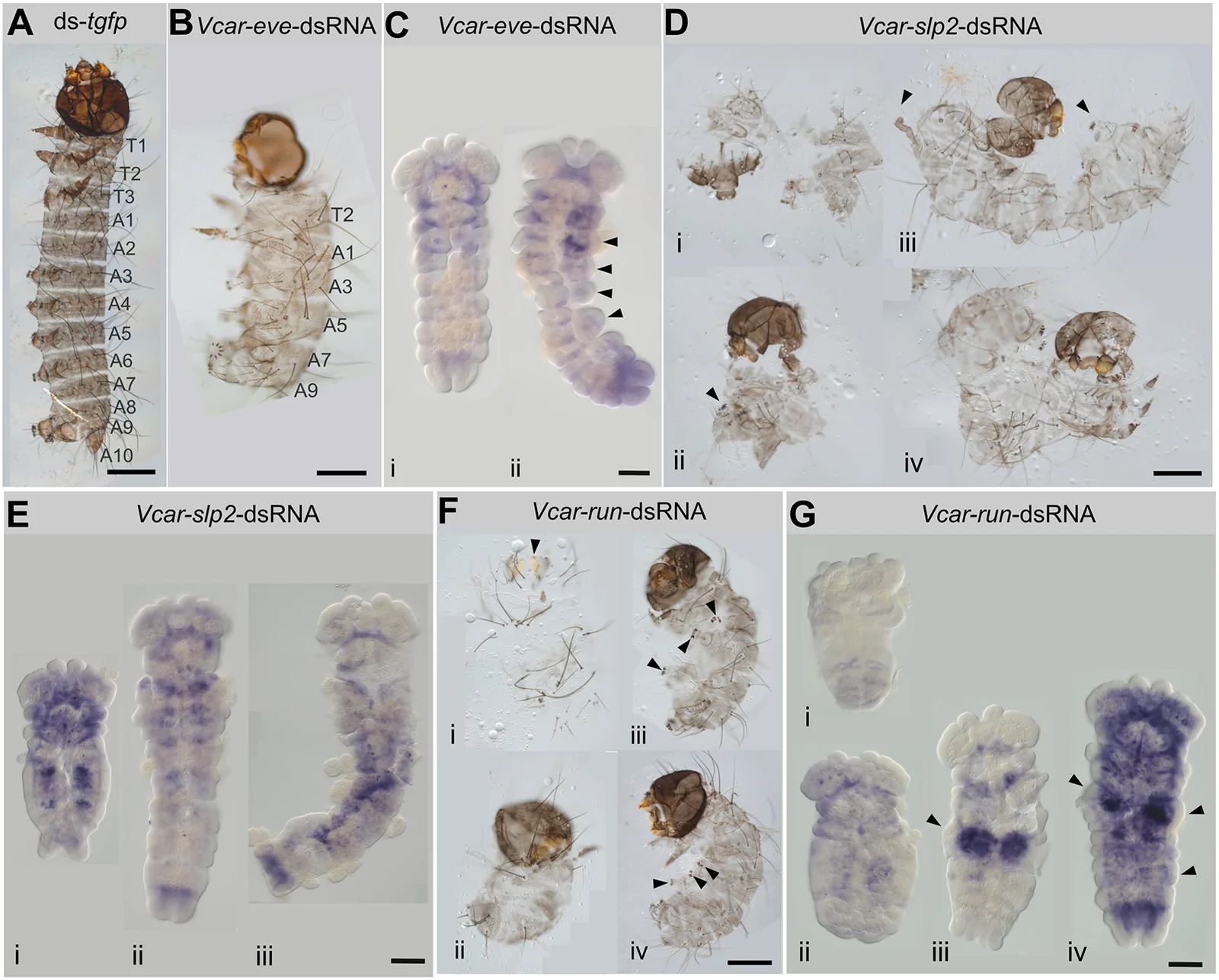
***Vanessa* PR gene orthologs function in segmentation.** (A-G) Cuticle preparations of eRNAi samples (A,B,D,F) and *Vcar-en* expression 24 h post-injection (C,E,G). (A) *tgfp*-dsRNA eRNAi embryos appeared wild type-like. (B,C) In *Vcar-eve* eRNAi samples (B) alternate segments (A2, A4, A6, A8, A10) were lost. (C) Loss of segments and partial fusions (arrowheads) in germbands. (D) Examples of *Vcar-slp2* eRNAi: (i) head-like; (ii) head and prolegs (arrowhead); (iii) head, legs (arrowhead) and prolegs (arrowhead); (iv) torus. (E) Examples of embryos lacking defined segments (i) and with developed germbands with non-specific *Vcar-en* expression (ii,iii). (F) *Vcar-run* eRNAi cuticle defects included: (i) undefined mass with defined mandible (arrowhead); (ii) undefined mass with head capsule; (iii,iv) head, one leg and two or three prolegs (arrowheads). (G) (i) *Vcar-en* expression was faint and many germbands had undefined shapes; (ii) defined head structures but undefined segments; (iii,iv) fused segments (arrowheads). Images orientated anterior top, posterior bottom. Scale bars: 200 µm (A,B,D,F); 100 µm (C,E,G).

In *Vcar-eve* RNAi embryos missing segments, *Vcar-en* expression localized in the stomodeum, gnathal segments and telson (Fig. 4Ci). For embryos with partial segmental fusions, *en* was detected in the stomodeum, each partial segment, and the telson (Fig. 4Cii). In knockdown germbands, we observed expression in the stomodeum, thoracic segments and telson, areas in which *en* is not normally expressed. Overall, although *Vcar-en* expression was clearly altered after *Vcar-eve* knockdown, we did not capture a stage at which PR-like loss of alternate *en* stripes was observed.

### *Vanessa slp2* affects segmentation

We isolated two *Vanessa sloppy paired* genes, *slp1* and *slp2*, each of which is expressed in a striped manner (Fig. 3D,E). After *Vcar-slp1* dsRNA1 injection, while most embryos were wild type-like (84%, *n*=475/564), some displayed delayed development (7%, *n*=39/564) or no development (9%, *n*=50/564). A second, non-overlapping dsRNA produced similar results (Table S1, Fig. S4B).

*Vcar-slp2* eRNAi produced a range of defects, with five distinct phenotypes observed in 64% (*n*=183/284) of dsRNA1-injected embryos and 35% (*n*=159/458) of dsRNA2-injected embryos (details in Table S1). The rest were wild type-like, displayed delayed development, or failed to develop (36%, *n*=101/284 for dsRNA1; 65%, *n*=299/458 for dsRNA2; Table S1). Three phenotypes were similar to those we observed for *Vcar-gsb* eRNAi: (1) head-like (Fig. 4Di), (2) head and one set of prolegs (Fig. 4Dii), and (3) torus (Fig. 4Div). Other phenotypes included (4) head, legs and prolegs with the head capsule displaying wild type-like stemmata, frons and antenna, one or two thoracic segments with leg-like structures, and five to six abdominal segments with prolegs (Fig. 4Diii, arrowheads) and (5) a variety of partial fusions, with thoracic segments mainly affected. For example, two fused thoracic segments with three legs and wild type-like abdominal segments (Fig. S3Bi, asterisks indicating legs). Others displayed all thoracic segments, with two legs, and the first three abdominal segments fused (Fig. S3Bii). Lastly, some displayed thoracic segments without legs, and some fused abdominal segments producing loss of prolegs, often with only two or three prolegs present (Fig. S3Biii).

*Vcar-en* expression in *Vcar-slp2* dsRNA2-injected embryos revealed (1) very short germbands with defined head lobes but no segmental grooves and a V-shaped telson (Fig. 4Ei); and (2) head lobes, fused gnathal, thoracic and first abdominal segments with defined telson and different degrees of fused segments in other parts of the embryo (Fig. 4Eii,Eiii). *Vcar-en* expression was not uniform across these germbands, with stripes in some segments.

In *Drosophila*, *slp1* and *slp2* expression patterns are similar and have a redundant functional relationship in segmentation (Grossniklaus et al., 1992; Cadigan et al., 1994). We therefore tested whether knockdown of both *Vcar-slp1* and *Vcar-slp2* would result in more severe segmentation phenotypes than either alone. Almost half (∼48%, *n*=209/433) of the embryos presented the five key *Vcar-slp2* phenotypes (Table S4). We did not observe more severe phenotypes for the mix of *Vcar-slp1* and *Vcar-slp2* dsRNAs, suggesting that *Vcar-slp2* is the main *slp* that functions in segmentation in the butterfly, but no PR-like function was seen.

### *Vanessa run* has a role in segmentation

To knock down *Vcar-run*, we designed two non-overlapping dsRNAs. dsRNA1 targets the region encoding the Runt domain; dsRNA2 targets the 3′ UTR (Fig. S1G). *Vanessa* embryos injected with *Vcar-run* dsRNA showed three main phenotypes (72%, *n*=446/617, of dsRNA1-injected embryos; 53%, *n*=333/624, of dsRNA2-injected embryos; Table S1). The remaining prehatchlings (28%, *n*=171/617, of dsRNA1-injected embryos; 47%, *n*=291/624, of dsRNA2-injected embryos) appeared wild type-like or displayed no development (Table S1). The most common segmentation phenotypes were: (1) Undefined mass of undeveloped tissue. In rare cases, it was possible to identify structures – mandibles (Fig. 4Fi, arrowhead) or a head capsule with stemmata, mandibles and shortened body (Fig. 4Fii) – within this mass. (2) Head that appeared wild type-like, one thoracic segment with one set of legs, and five or six abdominal segments with two or three sets of prolegs (Fig. 4Fiii,Fiv). The inconsistent presence of prolegs makes this phenotype difficult to classify as PR-like or not. (3) Partial segment fusions mainly in the thoracic region. In some cases, we observed two thoracic segments with legs and one without legs, and wild type-like abdominal segments (Fig. S3Ci). In others, we observed fusions of thoracic segments and the presence of leg-like structures; in some cases, fusions included the first abdominal segments with prolegs present (Fig. S3Cii,Ciii).

*Vcar-en in situ* hybridization in *Vcar-run* dsRNA-injected embryos revealed low expression of *Vcar-en* (Fig. 4Gi). In some cases, we observed diffuse *Vcar-en* expression in germbands with defined head structures and no segmental grooves (Fig. 4Gii). In others, strong or broad *Vcar-en* expression was observed in germbands with defined head structures, fused thoracic segments (Fig. 4Giii,Giv, arrowhead) and some defined abdominal segments. Overall, most *Vcar-run* knockdown embryos displayed defects in thoracic and abdominal segment formation. Because it was difficult to determine the register of segmental deletions and fusions, we were unable to definitively determine whether *Vcar-run* has PR-like function but its striped expression pattern is consistent with a PR role.

### An *Oncopeltus* PR-gene ortholog plays a novel role in *Vanessa*

In *Oncopeltus*, none of the *Drosophila* PR-gene orthologs plays a role in PR patterning (Reding et al., 2019), but *Oncopeltus* still follow a pair ‘rule’ with genes *E75a* (Erezyilmaz et al., 2009) and *Blimp1* (Reding et al., 2024) having PR expression and function. *Vcar-E75* expression was not detected in early embryos (Fig. 5Ai-Aiii). Once the germband started to elongate, faint expression appeared at the stomodeum and midline (Fig. 5Aiv,Av). *Vcar-Blimp1* expression was detected as wide bands in the blastoderm, one in the anterior region and the second in the posterior region (Fig. 5Bi, arrowheads), then in the germ primordium as two anterior bands while the posterior band of expression remained (Fig. 5Bii, arrowheads). Once the germband started to elongate, low expression in the protocephalon and three stripes in the posterior region were observed (Fig. 5Biii, arrowheads). During germband elongation, two widely spaced stripes were observed anterior to the SAZ (Fig. 5Biv, arrowheads). Later, expression localized to the stomodeum and midline region of posterior abdominal segments (Fig. 5Bv, bracket).

**Fig. 5.**
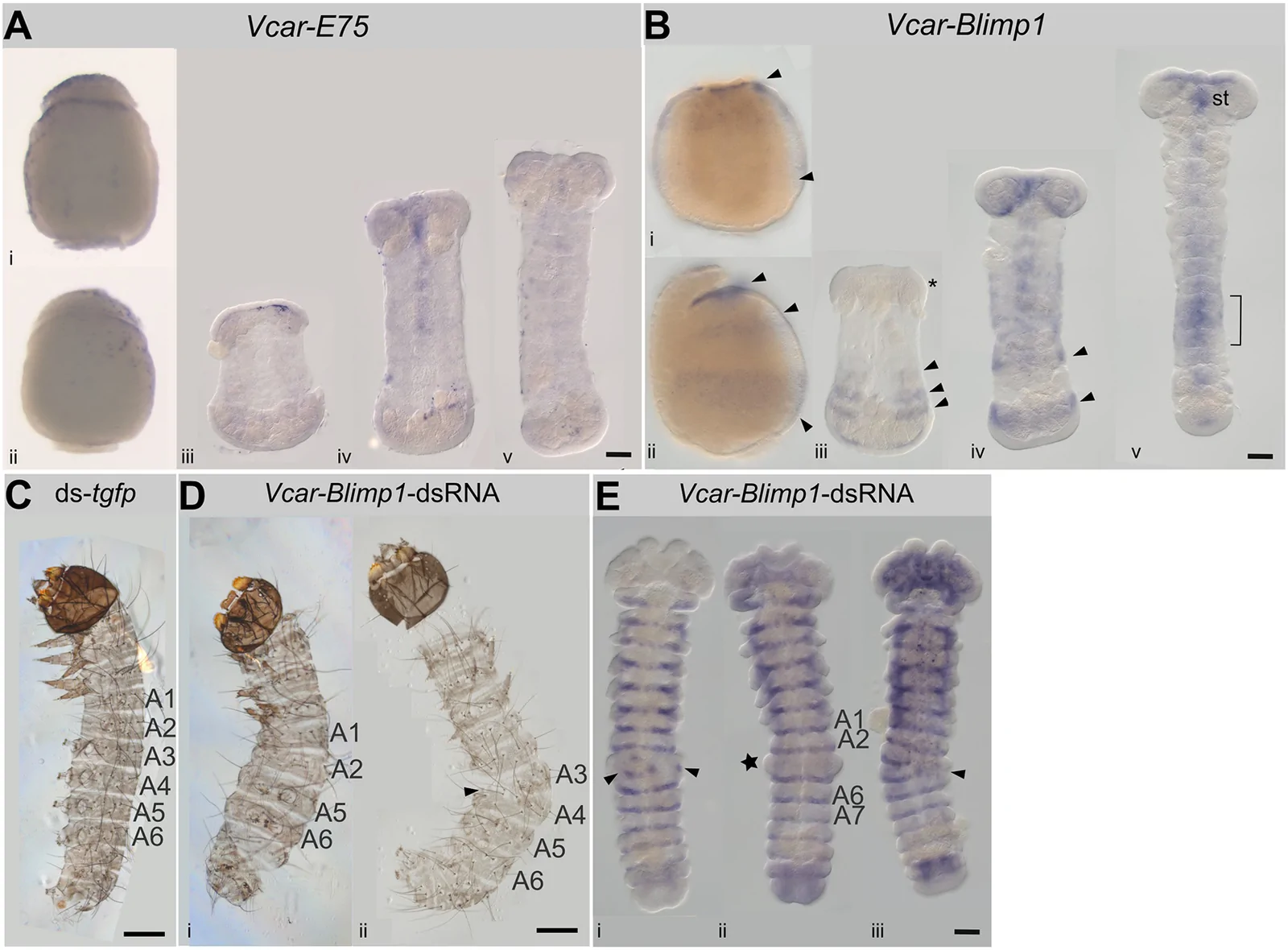
***Vcar-Blimp1* plays a novel role in segmentation.** (A) *Vcar-E75* expression was not detected in blastoderm (i) or germ primordium (ii). *Vcar-E75* expression was marginally detectable during germband elongation (iii-v). (B) *Vcar-Blimp1 in situ* hybridization: (i) one anterior and one posterior band in blastoderm (arrowheads); (ii) two anterior and one posterior band in germ primordium (arrowheads); (iii) weak expression in head lobes (asterisk) and three stripes (arrowheads) anterior of the SAZ; (iv) midline expression, two widely spaced stripes (arrowheads); (v) strong expression in abdominal segment primordia (bracket). (C) *Vanessa tgfp*-dsRNA eRNAi. Cuticular preparations were wild type-like; segments A1-A6 indicated. (D) *Vcar-Blimp1* eRNAi cuticular preparations: (i) loss of abdominal segments A3 and A4 or (ii) partial fusions of A3 and A4 (arrowhead). (E) *Vcar-en* expression in *Vcar-Blimp1* eRNAi embryos. (i) Germbands with fusion of segments (arrowheads); (ii) completely fused segments A3-A4 (star); (iii) partially fused A3-A4 segments (arrowhead). Images orientated anterior top, posterior bottom. Scale bars: 200 µm (C,D); 100 µm (A,B,E).

We performed *Vcar-E75* eRNAi experiments with two non-overlapping dsRNAs. *Vcar-E75* dsRNA1 and dsRNA2 match and extend slightly beyond the 5′ and 3′ portions, respectively, of the region encoding the ligand binding domain (Fig. S1K). After injection of *Vcar-E75*-dsRNA, most embryos appeared wild type-like (dsRNA1: 93%, *n*=569/611; dsRNA2: 78%, *n*=244/312). A small number displayed delayed development or no development (see Table S1), similar to numbers seen for controls (Fig. S4C). Thus, we found no evidence that *Vcar-E75* plays a role in segmentation.

*Vcar-Blimp1* knockdown was performed with two non-overlapping *Vcar-Blimp1* dsRNAs, dsRNA1 matching the region encoding the SET domain and dsRNA2 targeting a portion of the region encoding the zinc fingers (Fig. S1L). Most embryos injected with *Vcar-Blimp1*-dsRNA showed complete or partial loss of segments A3 and A4, with no other segmental defects apparent (Fig. 5Di; dsRNA1: 79%, *n*=431/545; dsRNA2: 56%, *n*=247/439; Table S1). In other cases, partial fusions of A3+A4 were observed on one side of the prehatchling but not the other, allowing us to count and definitively identify which segments were affected (Fig. 5Dii, arrowhead). The remaining prehatchlings (dsRNA1: 21%, *n*=114/545; dsRNA2: 44%, *n*=192/439) appeared wild type-like or displayed no development (Table S1).

*Vcar-Blimp1* dsRNA-injected embryos displayed *Vcar-en* expression in every segment. In some cases, fusions of segments A3+A4 were observed (Fig. 5Ei, arrowheads). Some germbands showed a bulging segment between segments A2 and A6 (Fig. 5Eii, star). This enlarged segment appeared to fuse A3+A4+A5, based on germbands that showed fusion on one side of the embryo but not the other (Fig. 5Eiii, arrowhead). These results suggest that *Vcar-Blimp1* functions in segmentation in a non-PR manner in *Vanessa*.

## DISCUSSION

Here, we described the early embryonic development of *V. cardui* and expression patterns of *Vanessa gsb* and orthologs of the *Drosophila* and *Oncopeltus* PR genes (Figs 1, 2, 3, 5). Except *Vcar-ftz-f1*, the orthologs of all *Drosophila* PR genes were expressed from blastoderm stage to germband elongation in *Vanessa*. eRNAi knockdown suggests segment polarity-like function for *Vcar-gsb* and *Vcar-slp2*, and classic PR function for *Vcar-eve. Vcar-run* knockdown embryos displayed fusion or loss of some or all segments, possibly indicative of PR function. Knockdown of *Vcar-Blimp1* produced fusions of abdominal segments A3 and A4 (Figs 2, 4, Fig. S3, Fig. 5), possibly indicative of gap-like function, although gap-like expression was not observed. Thus, some *Vanessa* segmentation genes share functions seen in *Drosophila* and other holometabolous insects.

### *gsb* has segment polarity-like function in *Vanessa*

We showed previously that the *Pax3/7* family member *prd*, a PR gene in *Drosophila*, is absent in Lepidoptera. However, we identified the presence of two genes from the *Pax3/7* family, *gsb* and *gooseberry neuro* (Gutiérrez Ramos and Pick, 2025). Because *gsb* has replaced *prd* in mosquitoes (Cheatle Jarvela et al., 2020), we investigated *gsb* expression and function in more detail in Lepidoptera. *Bicyclus gsb* and *Vanessa gsb* are both expressed in late embryos in the mandibular segment (Matsuoka et al., 2023; Gutiérrez Ramos and Pick, 2025), similar to the localization of *prd* in the mandibular and maxillary segments in *Tribolium* (Coulcher and Telford, 2012). *Bicyclus gsb* expression was also observed in the prolegs of late embryos; however, we did not assess this stage in *Vanessa*. *Vcar-gsb* is expressed in segmental stripes prior to and during germband formation (Fig. 2), like segment polarity genes *wingless* (*wg*) and *en* in other lepidopterans (Kraft and Jäckle, 1994; Xu et al., 1997; Nakao, 2010; Holzem et al., 2019; Hanly et al., 2021; Patel et al., 1989; Nagy and Carroll, 1994). In *Bombyx*, *wg* knockdown produces fused or truncated abdominal segments, and in some cases the phenotype was similar to our *Vcar-gsb* dsRNA torus phenotype (Yamaguchi et al., 2011; Zhang et al., 2015). In our functional experiments, *Vcar-gsb* knockdown also produced severely truncated embryos, some composed primarily of head tissue and others with fused segments (Fig. 2), similar to the head-only phenotypes seen for PR gene knockdown in beetles (Choe et al., 2006; Xiang et al., 2017). Therefore, while we can conclude that *Vanessa gsb* plays a role in every segment, we are unable to rule out the possibility that it also has an earlier PR-like function.

### PR gene expression patterns and function in other Lepidoptera

*Vcar-eve* and *Vcar-run* expression patterns appear similar to those reported for their orthologs in *Bombyx. Bombyx-eve* and *Bombyx-run* are both expressed in PR-like stripes (Nakao, 2010, 2015; Liu et al., 2008). *Bombyx-eve* eRNAi results in an asegmental phenotype (Xu et al., 1997; Nakao, 2010), while we found classic PR-like loss of alternate body segments for *Vcar-eve* RNAi. *Bombyx-run* RNAi produces shortened abdomens (Liu et al., 2008; Nakao, 2015), similar to *Vcar-run*. Thus, lepidopteran *eve* and *run* appear to share PR roles with *Drosophila*. *Bombyx-odd* shows an expression pattern similar to *Bombyx-eve* and knockdown results in head-like phenotypes (Nakao, 2015), with abnormal numbers of thoracic and abdominal segments, or loss of segments (Yamaguchi et al., 2011). While we did not test the function of *Vcar-odd*, its expression pattern is consistent with PR-like function.

The role of Lepidopteran *E75* orthologs during embryonic development had not been examined previously. Our results suggest that it does not play a role in segmentation in *Vanessa*. Lastly, *Bombyx-Blimp1* expression and function have been characterized in pupae and adults but no role in embryonic development has been reported (Wu et al., 2019). It will be interesting to see whether the *Vcar-Blimp1* role in abdominal segmentation also applies to other butterflies and moths.

### Lepidopteran PR gene orthologs within a broader context

Fig. 6 summarizes gene expression and function in the dipteran *Drosophila*, three lepidopterans (*Bicyclus*, *Bombyx* and *Manduca*), two coleopterans (*Tribolium* and *Dermestes*), two hymenopterans (*Nasonia* and *Apis*) and the non-holometabolous hemipteran *Oncopeltus* for the 13 genes that have PR function in at least one species, and two segment polarity genes, *wg* and *en*, which are expressed segmentally in all insect species examined (see references in Table S5).

**Fig. 6.**
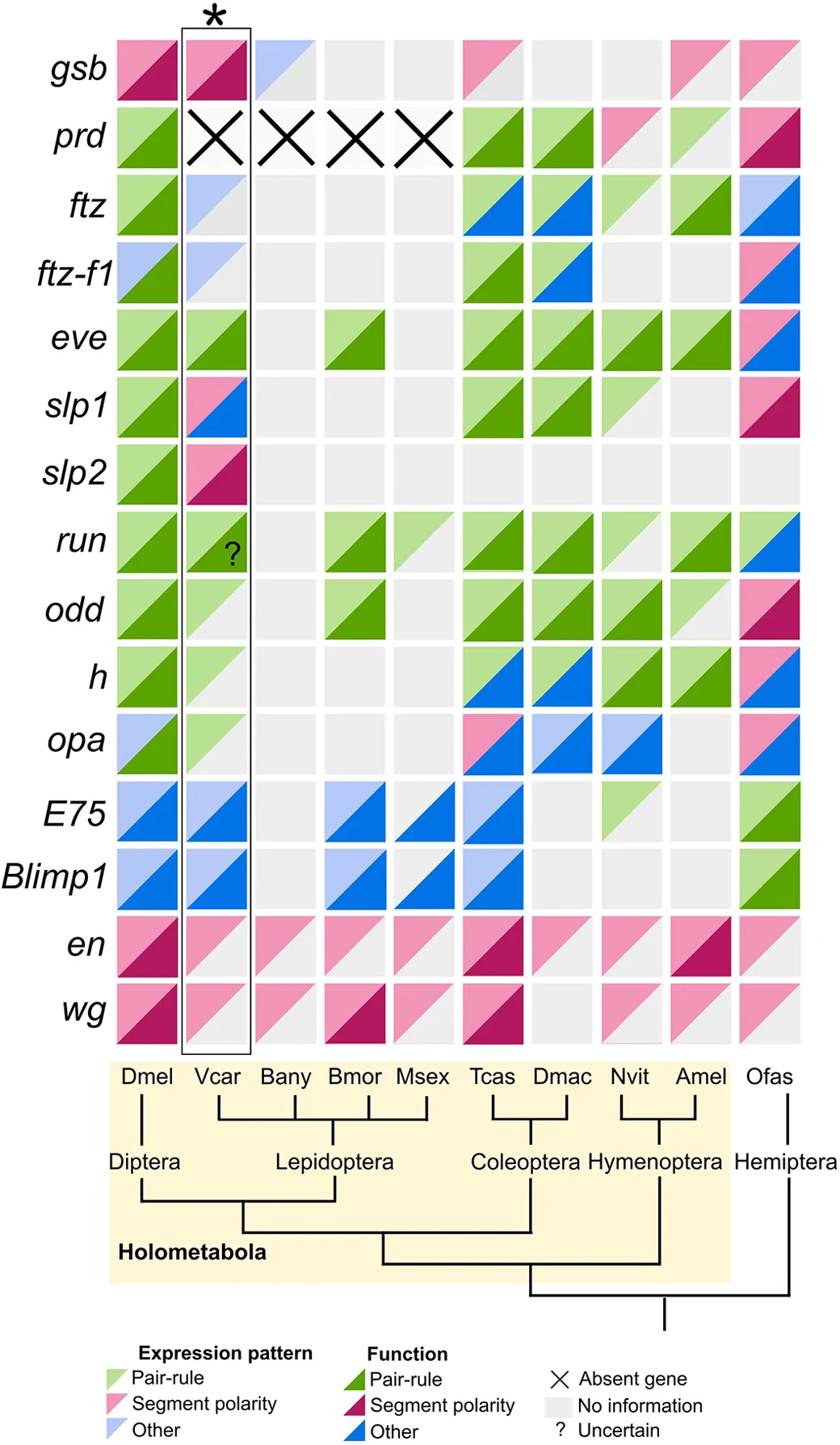
**Variation in expression and function of insect segmentation genes.** Summary of expression patterns and function of segmentation genes in selected species as indicated. Left: PR and segment polarity genes (*wg* and *en*). Matrix: Each row presents information about the expression pattern (light color) and/or the function of each gene (dark color) represented as triangles. Gray triangles indicate no information is available and X indicates absent genes. Each column represents insect species organized based on phylogenetic relationships (bottom): Amel, *Apis mellifera*; Bany, *Bicyclus anynana*; Bmor, *Bombyx mori*; Dmac, *Dermestes maculatus*; Dmel, *Drosophila melanogaster*; Msex, *Manduca sexta*; Nvit, *Nasonia vitripennis*; Ofas, *Oncopeltus fasciatus;* Tcas, *Tribolium castaneum*; Vcar, *Vanessa cardui* (present work, asterisk and boxed column). Of the insect species used, all species are from the order Holometabola except *Oncopeltus fasciatus* (Hemiptera), an outgroup in which these genes have been studied. We note that we cannot rule out, for our studies and likely for published work of others as well, short periods of transient expression missed due to embryo staging. See Table S5 for a summary of publications used to compile these data.

In *Vanessa*, we showed that *gsb* is expressed segmentally and has segment polarity-like function, similar to *Drosophila* (Nüsslein-Volhard and Wieschaus, 1980), *Tribolium* (Aranda et al., 2008), *Apis* (Osborne and Dearden, 2005) and *Oncopeltus* (Reding et al., 2019). We previously confirmed the loss of *prd* in Lepidoptera (Gutiérrez Ramos and Pick, 2025). *prd* functions as a PR gene in *Drosophila* (Nüsslein-Volhard and Weisman, 1980; Kilchherr et al., 1986), *Tribolium* (Maderspacher et al., 1998; Choe and Brown, 2007), *Dermestes* (Xiang et al., 2015) and *Apis* (Osborne and Dearden, 2005). However, in *Nasonia* and *Oncopeltus*, *prd* expression and function is segment polarity-like (Keller et al., 2010; Reding et al., 2019). *Vcar-ftz* is expressed as wide bands in the blastoderm, similar to *Tribolium* (Brown et al., 1994). *ftz* is expressed in a PR manner in *Tribolium*, *Dermestes*, *Nasonia* and *Apis* (Wilson and Dearden, 2012; Heffer et al., 2013; Xiang et al., 2017; Taylor and Dearden, 2022), but is not required for PR patterning in *Tribolium* or *Dermestes*. In *Vanessa* and all insects examined to date, *ftz* is expressed in the developing nervous system (Doe et al., 1988; Heffer et al., 2013; Reding et al., 2019). The expression of *Vcar-ftz-f1* in early embryos expression was not similar to other insects: in *Tribolium* and *Dermestes* the expression is PR (Heffer et al., 2013; Xiang et al., 2017) while it is segmental in *Oncopeltus* (Reding et al., 2019).

*eve* is the only gene with PR expression and function across holometabolous insects examined to date, although more studies have examined *eve* than other PR-gene orthologs. In *Drosophila*, *Bombyx*, *Tribolium*, *Dermestes*, *Apis* and *Nasonia, eve* is expressed in PR stripes and knockdown results in the loss of alternate segments (Nüsslein-Volhard and Wieschaus, 1980; Macdonald et al., 1986; Patel et al., 1994; Binner and Sander, 1997; Xu et al., 1997; Choe et al., 2006; Nakao, 2010; Wilson et al., 2010; Wilson and Dearden, 2012; Rosenberg et al., 2014; Xiang et al., 2017; Taylor and Dearden, 2022). However, in *Oncopeltus*, like other *Drosophila* PR orthologs, *eve* is expressed segmentally and functions segmentally (Liu and Kaufman, 2005). Both *Vcar-slp1* and *Vcar-slp2* are expressed segmentally in the blastoderm, and *slp2* has segment polarity-like function. These observations contrast with the PR expression of *slp* in *Drosophila*, *Tribolium*, *Dermestes* and *Nasonia*.

Similarly, PR expression for *run* is shared by many holometabolous insects, having been documented for *Drosophila*, *Bombyx*, *Manduca*, *Tribolium*, *Dermestes*, *Nasonia* and *Apis* (Gergen and Butler, 1988; Kraft and Jäckle, 1994; Choe et al., 2006; Liu et al., 2008; Wilson and Dearden, 2012; Rosenberg et al., 2014; Nakao, 2015; Xiang et al., 2017; Taylor and Dearden, 2022). In the hemipteran insect *Oncopeltus*, *run* was the only *Drosophila* PRG ortholog with an expression pattern resembling PR, although RNAi failed to reveal PR function (Reding et al., 2019). Our functional experiments in *Vanessa* revealed a possible PR-like loss of alternate segments, as well as a severe knockdown phenotype with only head-like structures, similar to *run* knockdown phenotypes in *Tribolium*, *Dermestes* and *Bombyx* (Choe et al., 2006; Liu et al., 2008; Xiang et al., 2017).

Two other genes that have PR expression in many holometabolous insects are *odd* and *h*. For *odd*, both expression and function in *Drosophila*, *Bombyx*, *Tribolium*, *Dermestes* and *Nasonia* have been reported as PR (Coulter et al., 1990; Choe et al., 2006; Yamaguchi et al., 2011; Rosenberg et al., 2014; Nakao, 2015; Xiang et al., 2017; Taylor and Dearden, 2022). However, changes have been observed for *h*, which has PR function in *Drosophila*, *Nasonia* and *Apis* but not in either *Tribolium* or *Dermestes* (Ingham et al., 1985; Sommer and Tautz, 1993; Aranda et al., 2008; Wilson and Dearden, 2012; Rosenberg et al., 2014; Xiang et al., 2017; Taylor and Dearden, 2022).

*Drosophila opa* functions as a PR gene (Benedyk et al., 1994); however, *opa* has shown variability in its early embryonic expression pattern and function among different insects. In *Vanessa*, *opa* is expressed in segmental stripes, whereas in *Drosophila* it is expressed ubiquitously (Benedyk et al., 1994; Clark and Akam, 2016). In *Tribolium* and *Oncopeltus, opa* is expressed segmentally; but no segmentation function has been identified in *Oncopeltus* (Reding et al., 2019) and its function is unclear in *Tribolium* (Choe et al., 2017; Clark and Peel, 2018). In *Dermestes* and *Nasonia,* neither the expression nor function of *opa* is PR (Xiang et al., 2017; Taylor and Dearden, 2022).

*E75a* and *Blimp1* are PR genes in *Oncopeltus* but little is known about their roles in embryonic development in holometabolous insects. *Nasonia E75a* displays a PR expression pattern (Taylor and Dearden, 2022), but we did not observe a clear pattern in the butterfly. While not PR, we observed a segmentation role for *Vcar-Blimp1* in abdominal segments A3 and A4. How *Blimp1* fits into a segmentation gene network in butterflies remains to be investigated.

In conclusion, the expression and function of some genes important for segmentation in insects are conserved in a butterfly model system. *Vanessa-gsb* appears to share with *Drosophila* a role as a segment polarity gene. While several of the orthologs of *Drosophila* PR genes have PR expression and/or function in *Vanessa*, others do not*. Vanessa* does not ‘make up for this deficit’ by utilizing orthologs of the *Oncopeltus* PR genes for PR patterning. Interestingly, *Vcar-Blimp1* plays a different role in segmentation, being required for the formation of only two abdominal segments. Thus, this gene has changed function more than once during insect evolution, functioning as a PR gene in *Oncopeltus* but playing no role in segmentation in *Drosophila.*

## MATERIALS AND METHODS

### *Vanessa* rearing and embryo collection

The *Vanessa* colony was maintained in an incubator (Percival) at 25°C with a 12-h light/dark cycle at 50% humidity. Larvae were maintained on an artificial diet (Multiple Species Diet, Southland Products) and adults were fed with a 50% diluted sports drink (Gatorade:tap water). Five-day-old adults were exposed to a hollyhock plant for a defined period to collect embryos. Embryos were removed from the hollyhock leaves and grown in a Petri dish until the desired time at 25°C.

### *Vanessa* gene ortholog identification and isolation

The annotated *Vanessa* genome [PRJEB42869, ilVanCard2.1 (GCF_905220365.1)] was used to identify the *Drosophila* and *Oncopeltus* PR gene ortholog sequences using tBLASTn. Once *Vanessa* segmentation genes were identified, each protein domain was manually annotated, indicated in Fig. S1. Next, we selected and identified segmentation protein sequences from different arthropods (see Table S6) to perform phylogenetic reconstructions (Fig. S2). Sequence alignment used MAFFT v.7.511 (Katoh and Standley, 2013) with L-INS-i model and arguments: –phylipout –reorder. The maximum likelihood trees were built with IQ-Tree 3.1.2 (Wong et al., 2026), using the model Q.insect+I+R4, with 1000 ultrafast bootstraps (Hoang et al., 2018). Visualizations were carried out using ggtree v.3.23 (Yu et al., 2017) and Inkscape v.1.3.2.

For gene isolation, primers (Table S7) were designed using the transcript sequences identified from NCBI. For RT-PCR, total RNA was extracted from embryos 16 to 24 h AEL or from adult female posterior abdomens. For embryonic RNA, 100 µl of embryos were collected. Female posterior abdomens were used to extract adult female RNA. Total RNA was extracted using TRIzol (Invitrogen) per manufacturer instructions and DNase treated with TURBO DNA-free kit (Invitrogen). cDNA synthesis was carried out with NEB reagents using random primer mix. All PCR products were inserted into a pGEM vector (Promega) and Sanger sequenced.

### Embryo fixation and nucleic acid staining

Embryos used for nucleic acid staining were collected for 1-h periods for the first 8 h of development and an 8-h collection was performed to examine embryos from 8 to 16 h AEL. All embryos were allowed to develop in Petri dishes under the same conditions as adults until the desired timepoint. For embryo fixation, a tube of ∼300 µl of embryos and 500 µl of water was boiled for 3 min, then moved to ice for 10 min. The chorion was manually removed in a Pyrex spot plate in tap water. The embryos were then fixed with 4% paraformaldehyde in PBS with 0.1% Tween-20 (PBST) for 20 min by rocking, washed with PBST and dehydrated gradually into 100% methanol. Samples were stored at −20°C until use. For the nucleic acid stain, the embryos were rehydrated gradually into 100% PBST, then incubated at room temperature for 10 min with 50 µg/ml Hoechst 34580 in PBST with rocking. They were rinsed three times with PBST and mounted on slides in 70% glycerol/PBST. Embryos were imaged by differential interference contrast (DIC) with DAPI filter on a Zeiss Axio Imager M1 microscope and images were arranged with ImageJ v.1.54f (Schneider et al., 2012) and Inkscape v.1.3.2.

### Embryo gene expression analysis

Embryo collections and fixation were performed as in the ‘Embryo fixation and nucleic acid staining’ section except that, after boiling, embryos were washed with 1.5% bleach for 45 s, then were fixed by rocking in 4% paraformaldehyde in PBST for 20 min, washed with PBST and then dehydrated gradually into 100% methanol. Embryos were stored at −20°C until use. For *in situ* hybridization, embryos stored at −20°C were rehydrated into PBST and chorion and germbands were dissected from the yolk. The *in situ* hybridization protocol was carried out as described by Gutiérrez Ramos and Pick (2025). Embryo blastoderms were photographed with a Zeiss Discovery stereoscope in a Pyrex spot plate in PBST. Embryo germbands were mounted on a slide with 70% glycerol/PBST and photographed with DIC on a Zeiss Axio Imager M1 microscope; several images were taken for each embryo that were then stitched together using Adobe Photoshop and arranged with ImageJ v.1.54f (Schneider et al., 2012) and Inkscape v.1.3.2.

### eRNAi experiments

Synthesis of dsRNA and embryo microinjections 3 h AEL were performed as described by Gutiérrez Ramos and Pick (2025). Briefly, for each gene knockdown, we used two non-overlapping dsRNAs, which were injected and then observed phenotypes were scored (Table S1). For each gene, a chi-square statistical test was performed to compare dsRNA1 and dsRNA2 (Table S2), which showed that, for all genes, both dsRNAs were non-independent, and therefore both dsRNAs had a statistically significant association. For all eRNAi injections (Table S8), 1.5 µM dsRNA of each dsRNA was injected first. For most of the genes, we observed a segmentation phenotype using this concentration. For *Vcar-slp1* and *Vcar-E75*, we did not observe defects, so we increased the concentration to 3 µM to rule out the possibility that the dose of dsRNA was too low. However, we still observed wild type-like phenotypes (Table S1). For cuticle phenotype scoring, injected embryos were grown for 3 days, then boiled for 3 min, cooled and dissected from the chorion for examination. For imaging cuticles, individual larva were mounted on a slide, covered with 100 µl of lactic acid, coverslipped, incubated overnight at 60°C, and imaged by DIC on a Zeiss Axio Imager M1 microscope; several images taken for each larva were stitched together using Adobe Photoshop and arranged with ImageJ v.1.54f (Schneider et al., 2012) and Inkscape v.1.3.2. For gene expression pattern analysis, embryos were boiled, fixed and subjected to *in situ* hybridization 24 h post injection, as described above.

## Supplementary Material



10.1242/develop.205835_sup1 Supplementary information

Table S1. *Vanessa* PR-genes eRNAi phenotype counts.

Table S2. *Vanessa* pair-rule genes eRNAi phenotype Chi-Square analysis.

Table S3. *Vcar-gsb*-dsRNA1 phenotypes from dilution experiment.

Table S4. *Vanessa slp1* and *slp2* double knockdown eRNAi phenotypes.

Table S5. Insect segmentation gene references used for Figure 6.

Table S6. Segmentation protein sequences used in phylogenetic analysis.

Table S7. Primers used in this study.

Table S8. *Vanessa* PR-genes dsRNA sequences.
