# Supplementary material for: Segmentation gene expression and function in Vanessa cardui, an emerging model for Lepidoptera
Source: Development. 2026 Oct 12;153(19):dev205835. doi: 10.1242/dev.205835 (PMC13723396; doi:10.1242/dev.205835)
Supplement: Supplementary file 1 [file develop-153-205835-s1.pdf]

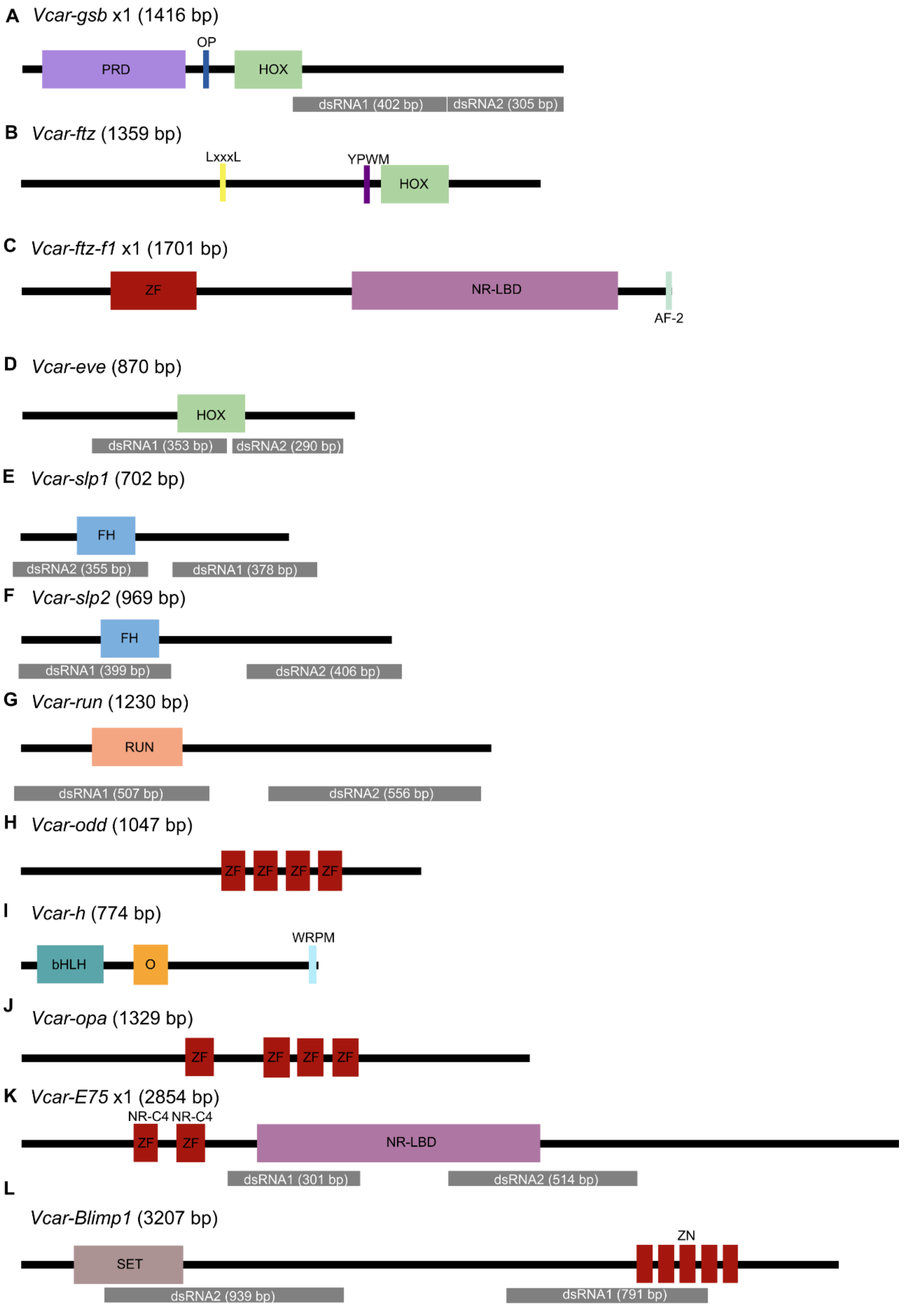

Development • Supplementary information

Fig. S1. *Vanessa* pair-rule genes structures. Schematic representation of the CDS of *Vanessa* PR-gene orthologs. Colored boxes denote protein domains and grey boxes under gene structures indicate the regions targeted by dsRNA. (**A**) *Vcar-gsb* isoform (x) 1 coding sequence (CDS) is 1416 base pairs (bp); Paired domain (PRD), Homeodomain (HOX) and an Octapeptide motif (OP) are shown. dsRNA1 is 402 bp; dsRNA2 is 305 bp. (**B**) *Vcar-ftz* complete cDNA is 2040 bp and its CDS is 1359 bp, encoding a complete HOX, as previously reported (Mulhair et al. 2023) and two motifs, LxxxL (LRAIL) and YPWM. dsRNA1 is 296 bp and dsRNA2 is 432 bp. (**C**) *Vcar-ftz-f1* complete cDNA is 8024 bp, with four predicted isoforms; their CDSs are *ftz-f1*-x1 with 1701 bp, *ftz-f1*-x2 with 1728 bp, *ftz-f1-x3* with 1686 bp, and *ftz-f1-x4* with 1626 bp. *Vcar-ftz-f1* encodes one zinc finger (ZF), a Ligand Binding Domain (indicated as NR-LBD) and the C-terminal AF-2. dsRNA1 is 785 bp and dsRNA2 is 463 bp. (**D**) *Vcar-eve* complete cDNA is 1045 bp. The 870 bp CDS encodes a HOX. dsRNA1 is 353 bp and dsRNA2 is 290 bp. (**E**) *Vcar-slp1* complete cDNA is 982 bp with a CDS 702 bp that encodes a Fork head (FH) domain. dsRNA1 is 355 bp and dsRNA2 is 378 bp. (**F**) *Vcar-slp2* complete cDNA is 1321 bp with a CDS 969 bp that encodes a FH domain. dsRNA1 is 399 bp and dsRNA2 is 406 bp. (**G**) *Vcar-run* complete cDNA is 1951; the 1230 bp CDS encodes a Runt (RUN) domain. dsRNA1 507 bp and dsRNA2 556 bp. (**H**) *Vcar-odd* complete cDNA is 1758 bp with a 1047 bp CDS that encodes four ZF domains located in the C-terminal half of the protein. (**I**) *Vcar-h* complete cDNA is 2035 bp; the CDS is 774 bp and encodes a basic helix–loop–helix (bHLH) domain, an Orange (O) domain and the WRPM motif in the carboxyl terminal. (**J**) *Vcar-opa* complete cDNA is 3560 bp and CDS is 1329 bp, that encodes four ZF domains. (**K**) *Vcar-E75* has four isoforms: *E75*-

x1 CDS is 2854 bp, *E75*-x2 CDS is 2537 bp, *E75*-x3 CDS is 2723 bp, and *E75*-x4 CDS is 2553 bp. *Vcar-E75* encodes two nuclear receptor C4-type (NR C4-type) ZF domains and a nuclear receptor ligand-binding (NR-LBD) domains. dsRNA1 301 bp and dsRNA2 514 bp. (**L**) *Vcar-Blimp1* complete cDNA is 5923 bp and has three isoforms: *Blimp1*-x1 CDS is 3207 bp, *Blimp1*-x2 CDS is 3165 bp and *Blimp1*-x3 CDS is 3207 bp. *Vcar-Blimp1* encodes a Su(var)3-9, Enhancer-of-zeste and Trithorax (SET) domain and five ZF domains. dsRNA1 is 939 bp and dsRNA2 is 791 bp.

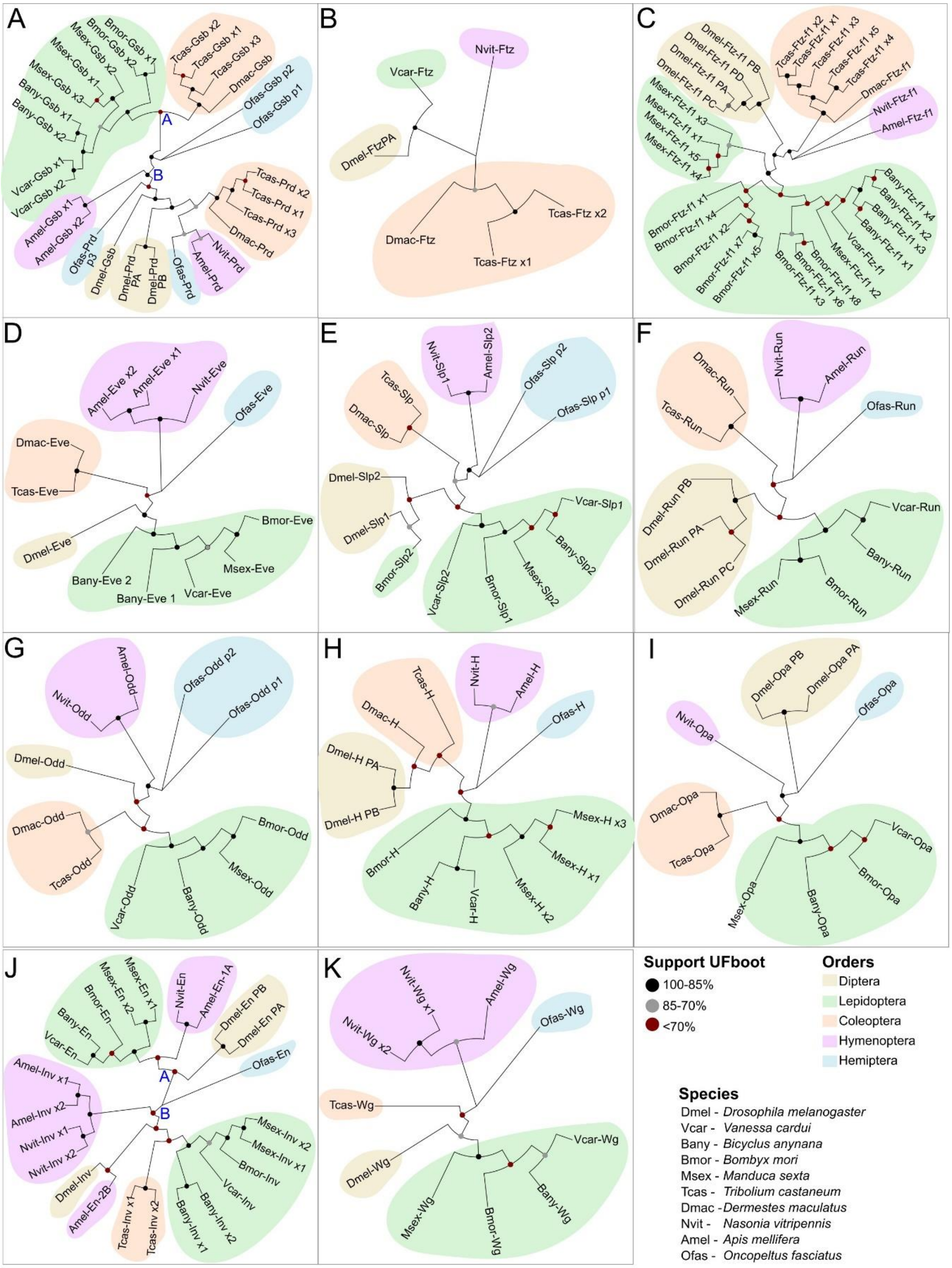

Gutiérrez Ramos et al. SuppFigure S2

Development • Supplementary information

Fig. S2. Phylogenetic analysis of *Vanessa* segmentation genes.

Maximum likelihood distribution of segmentation proteins from ten arthropods identified by their complete or partial protein sequence (see Table S1). (**A**). Gsb and Prd tree, we observed that are separated into two large clades, A where Gsb is located and B where Prd is located. We observed a similar distribution as previously reported in Gutierrez Ramos and Pick 2025, with the *Apis* Gsb sequences, that are in the Prd clade. (**B**). Ftz tree, we observe that *Vanessa* Ftz is in the same node as *Drosophila* Ftz. (**C**). Ftz-f1 tree, clear distribution of each arthropod order are in different nodes. (**D**). Eve tree, clear distribution of each arthropod order are in different nodes. (**E**). Slp1 and Slp2 tree, we observed that the distribution is not defined by one protein or the other, which may be because both proteins have not been identified in the selected arthropods. However, we observed that the lepidopteran proteins group at the same node. (**F**). Run tree, clear distribution of each arthropod order are in different nodes. (**G**). Odd tree, clear distribution of each arthropod order are in different nodes. (**H**). H tree, clear distribution of each arthropod order are in different nodes. (**I**). Opa tree, clear distribution of each arthropod order are in different nodes. (**J**). En and Invected (Inv) tree, we observed that are separated into two large clades, A where En is located and B where Inv is located. Localization of both proteins are as expected; however, *Apis* En-2B is group with *Drosophila* Inv. We did not find more information regarding the annotation of *Apis*-Inv, only the identification of two Apis En proteins (Walldorf et al. 1989). Therefore, we can suggest that Apis-En-2B could be Inv ortholog. (**K**). Wg tree, where a clear distribution of each arthropod order are in different nodes. Node support by UFboot (right down key) algorithms with 1,000 bootstrap value, both cutoffs for the node support are represented in colors. A black circle denotes a bootstrap valued 100-85%; a gray circle denotes a node support a bootstrap value 85-70%; a maroon circle denotes a bootstrap value <70%. Order species classifications

denotes by shade colors are Diptera – sand, Lepidoptera – green, Coleoptera – orange, Hymenoptera – pink, and Hemiptera – blue. Species name abbreviations are Dmel – *Drosophila melanogaster*, Vcar – *Vanessa cardui* (present work), Bany – *Bicyclus anynana*, Bmor – *Bombyx mori*, Msex – *Manduca sexta*, Tcas – *Tribolium castaneum*, Dmac – *Dermestes maculatus*, Nvit – *Nasonia vitripennis*, Amel – *Apis mellifera*, and Ofas – *Oncopeltus fasciatus*.

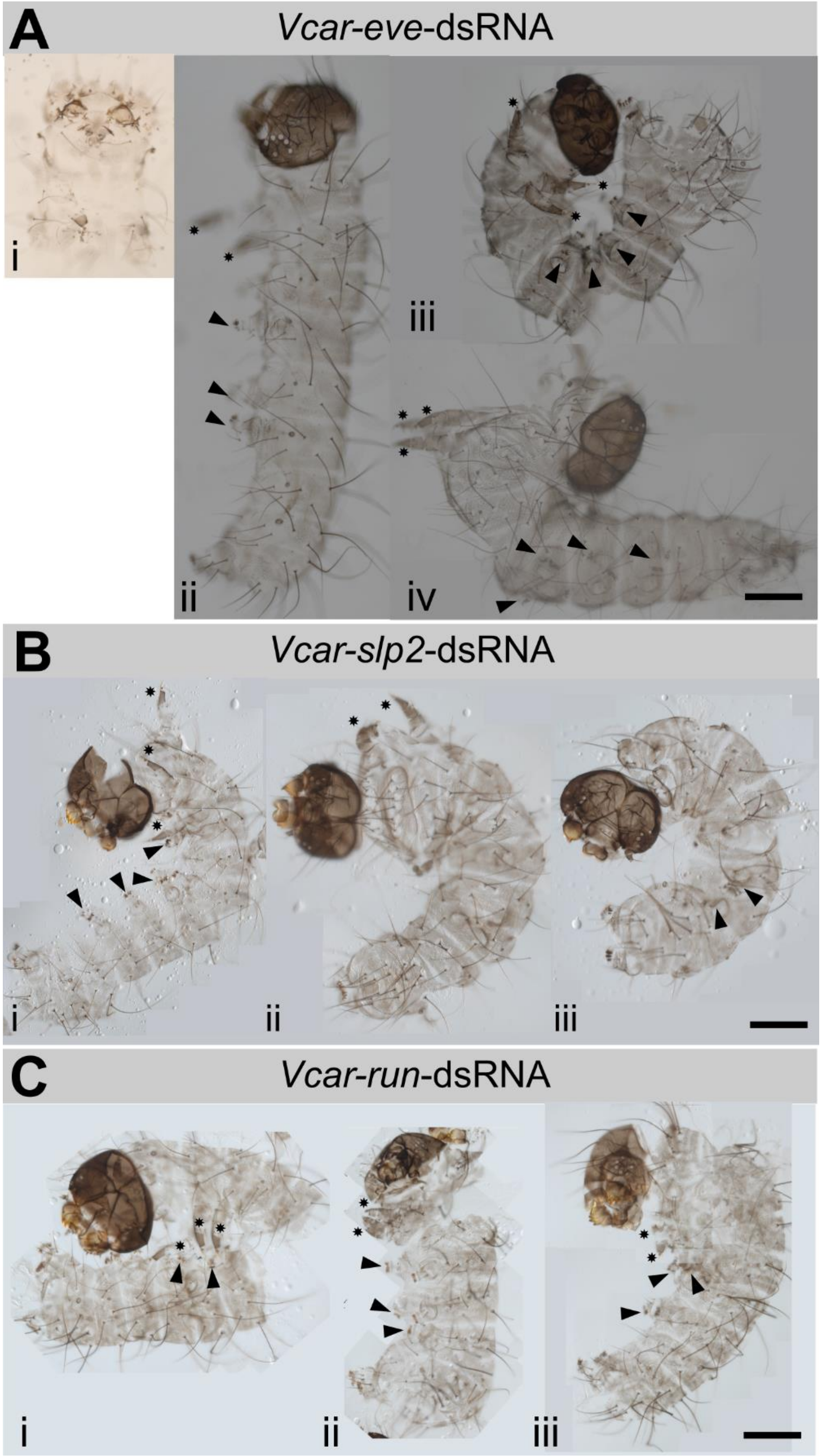

Development • Supplementary information

Fig. S3. ***Vcar-eve, Vcar-slp2*** **and** ***Vcar-run*** **partial fusion knockdown phenotypes.** Cuticle preparations of eRNAi embryos. (**A**) *Vcar-eve* knockdown phenotypes included head-like (i), and partial segmental fusions with the loss of two abdominal segments, presumably T1 and T3, leaving one thoracic segment with legs (dots) and the loss of one abdominal segment with one set of prolegs (arrowheads), presumably A4 (ii). Others present fusions of thoracic segments with the presence of three legs (dots) and wildtype-like abdominal segments (iii-iv). (**B**) For *Vcar-slp2* eRNAi knockdown, partial fusions mainly affected the thoracic segments. The most common phenotypes are: (i) two fused thoracic segments with three legs (stars) and wildtype-like abdominal segments, with the presence of 4 sets of prolegs (arrowheads); (ii) All thoracic segments, with just two legs (stars), and the first three abdominal segments fused; and (iii) thoracic segments present but lacking legs, and some abdominal segments fused that affected the presence of prolegs (arrowheads). (**C**) *Vcar-run* partial fusions mainly impacted thoracic segments. We observed the presence of two thoracic segments with their legs (stars) and one segment without legs with the rest of the abdominal segments wildtype-like arrowhead indicate prolegs (i). Others have thoracic segments fused with the presence of legs-like structures (stars) and fusions that cover the first abdominal segments; prolegs were present (arrowheads) (ii-iii). Images are orientated anterior, top; and posterior, bottom. Scale bar is 200 um.

Development • Supplementary information

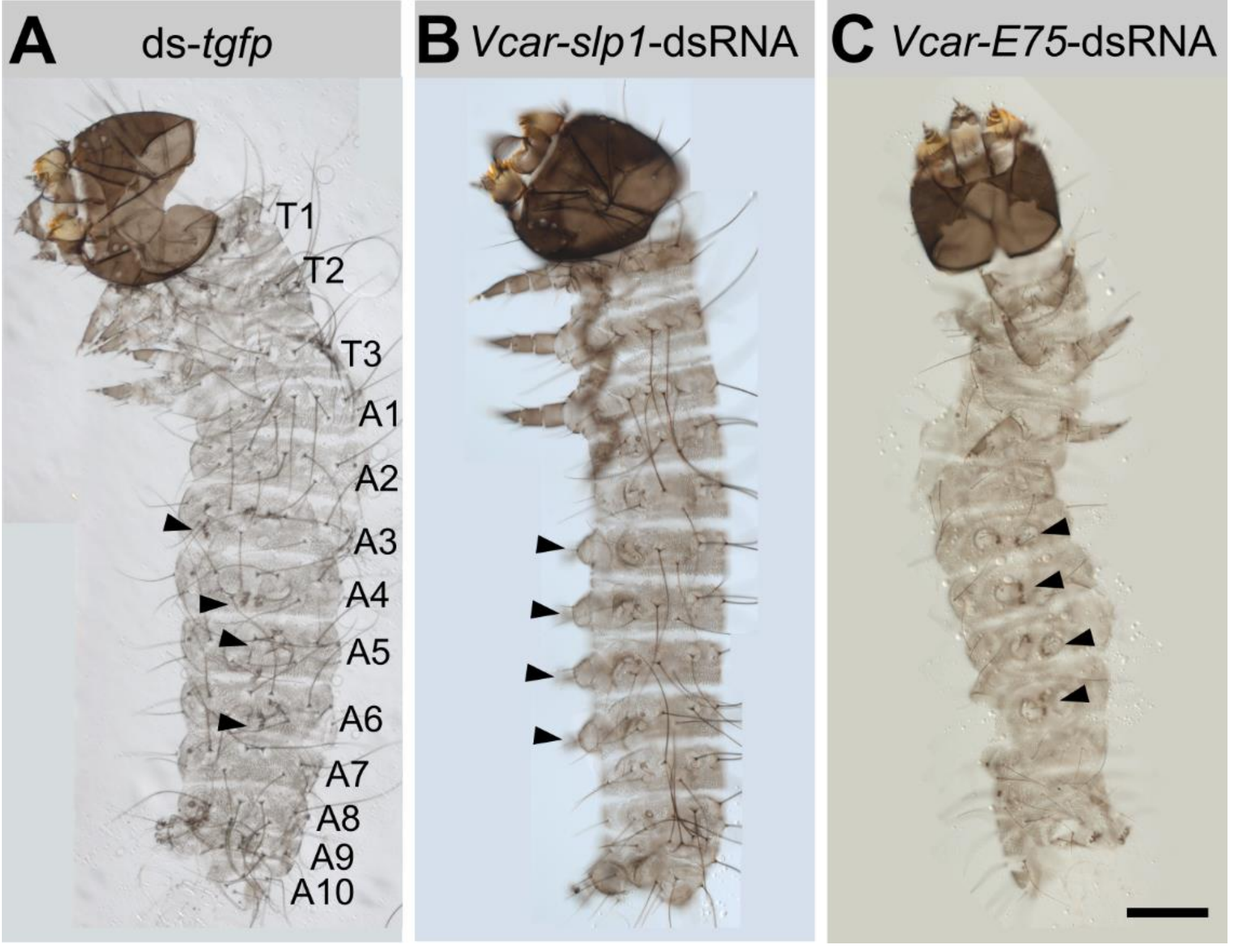

Fig. S4. Wildtype-like knockdown phenotypes for *Vcar-slp1* and *Vcar-E75*. Cuticle preparations of eRNAi embryos. (**A**) *tgfp*-eRNAi hatchlings are wildtype-like; the head, three thoracic segments (T1-T3) and ten abdominal segments (A1-A10) were all observed. A3-A6 and A10 have prolegs. (**B-C**) Wildtype-like phenotypes were observed after eRNAi targeting (**B**) *Vcar-slp1* or (**C**) *Vcar-E75*. Arrowheads show the prolegs. Images are orientated anterior, top; and posterior, bottom. Scale bar is 200 um.

Development • Supplementary information

Table S1. *Vanessa* PR-genes eRNAi phenotype counts.

Available for download at
https://journals.biologists.com/dev/article-lookup/doi/10.1242/dev.205835#supplementary-data

Table S2. *Vanessa* pair-rule genes eRNAi phenotype Chi-Square analysis. (A) Summary table of the Chi-Square analysis for all the *Vcar*-PR dsRNAs. **(B)** Chi-Square analysis observed and expected values for each dsRNA *Vcar*-PR gene.

Available for download at
https://journals.biologists.com/dev/article-lookup/doi/10.1242/dev.205835#supplementary-data

Table S3. *Vcar-gsb*-dsRNA1 phenotypes from dilution experiment.

Available for download at
https://journals.biologists.com/dev/article-lookup/doi/10.1242/dev.205835#supplementary-data

Table S4. *Vanessa slp1* and *slp2* double knockdown eRNAi phenotypes.

Available for download at
https://journals.biologists.com/dev/article-lookup/doi/10.1242/dev.205835#supplementary-data

Table S5. Insect segmentation gene references used for Figure 6.

Available for download at
https://journals.biologists.com/dev/article-lookup/doi/10.1242/dev.205835#supplementary-data

Table S6. Segmentation protein sequences used in phylogenetic analysis.

Available for download at
https://journals.biologists.com/dev/article-lookup/doi/10.1242/dev.205835#supplementary-data

Table S7. Primers used in this study.

Available for download at
https://journals.biologists.com/dev/article-lookup/doi/10.1242/dev.205835#supplementary-data

Table S8. *Vanessa* PR-genes dsRNA sequences.

Available for download at
https://journals.biologists.com/dev/article-lookup/doi/10.1242/dev.205835#supplementary-data
