# Supplementary material for: Segmentation gene expression and function in Vanessa cardui, an emerging model for Lepidoptera
Source: Development. 2026 Oct 12;153(19):dev205835. doi: 10.1242/dev.205835 (PMC13723396; doi:10.1242/dev.205835)
Supplement: Supplementary file 2 [file develop-153-205835_review_history.pdf]

Development

Segmentation gene expression and function in *Vanessa cardui*, an emerging model for Lepidoptera

Ximena Gutiérrez Ramos, Katie Reding and Leslie Pick
DOI: 10.1242/dev.205835

Editor: Cassandra Extavour

Review timeline
Original submission: 25 May 2026
Editorial decision: 6 July 2026
First revision received: 5 August 2026
Accepted: 21 August 2026

Original submission

First decision letter

MS ID#: dev.205835

MS TITLE: Segmentation gene expression and function in Vanessa cardui, an emerging model for Lepidoptera

AUTHORS: Ximena Gutiérrez Ramos; Katie Reding; Leslie Pick

Dear Dr Pick,

I have now received all the referees reports on the above manuscript, and have reached a decision. The referees' comments are appended below.

The overall evaluation is positive and we would like to publish a revised manuscript in Development, provided that the referees' comments can be satisfactorily addressed. Please attend to all of the reviewers' comments in your revised manuscript and detail them in your point-by-point response. If you do not agree with any of their criticisms or suggestions explain clearly why this is so. If it would be helpful, you are welcome to contact us to discuss your revision in greater detail. Please send us a point-by-point response indicating your plans for addressing the referees' comments, and we will look over this and provide further guidance.

Reviewer 1

SUMMARY OF THE ADVANCE MADE IN THIS PAPER AND ITS POTENTIAL SIGNIFICANCE TO THE FIELD

The paper takes a candidate gene approach to examine potential pair-rule (PR) expression and function in the butterfly, Vanessa cardui. The work is novel in investigating a butterfly (previous lepidopteran studies have focused on moths) and aside from documenting expression patterns of eleven orthologs of PR genes known from Drosophila and Oncopeltus (and the RNAi phenotypes of six), primary contributions include 1) a convincing demonstration that V. cardui even-skipped (Vcar-eve) is both expressed and functions as a pair-rule gene (Fig. 4B) and 2) the discovery that V. cardui Blimp1 plays a novel role in abdominal segmentation (Fig. 5D). These discoveries contribute to our overall picture of how the genetic and developmental mechanisms underlying a conserved morphology (in this case, segmentation) evolve.

© 2026. Published by The Company of Biologists under the terms of the Creative Commons Attribution License (https://creativecommons.org/licenses/by/4.0/).

SUGGESTIONS TO AUTHORS

While I found the evidence for PR expression and function for Vcar-eve (Figs. 3C and 4B) compelling—very cool how the lepidopteran prolegs helped here—I was less convinced by the evidence presented for V. cardui runt (Vcar-run) (Figs. 3F and 4F). I agree that the evidence is consistent with a possible PR role, but it did seem to fall short of being definitive on this point. As an easy remedy, here are several points in the introduction and discussion where the language could be qualified to bring it more in line with the stated results (e.g., Lines 324-325 and 337-338):

* Line 94: The use of "or" seems odd in this sentence. As expressed above, I also have some reservations about the PR claims for Vcar-run. Thus, consider: "…RNAi knockdown of Vcar-eve, and possibly Vcar-run, revealed a PR-like function."

* Line 223: Here there is mention of Vcar-run "stripes" in the posterior of the germband and in the SAZ. It may be the quality of the pdf I received, but I'm having a hard time seeing these stripes in Fig 3Fiii. If anything, I see broad expression throughout the SAZ, and even that's not entirely clear. Consider revisiting this description.

* Lines 382-383: Consider changing "…classic PR-function for Vcar-eve while revealed Vcar-run knockdown embryos displayed fusion or loss of some or all segments, likely indicative of PR-function." >> "…classic PR function for Vcar-eve, and a segmental role, possibly PR, for Vcar-run."

* Line 414: Add "possibly" so that it reads "…and possibly run appear…"

* Figure 6: Add a "?" to the PR function wedge in the square for Vcar-run to indicate some remaining uncertainty.

* Line 462: Change "likely" to "possible"

I realize that the difference between "likely" and "possible" is small and if you prefer the former, I suppose I would request that you explain why you believe a PR role is likely versus simply possible based on the evidence presented.

As described in the methods, Vcar orthologs were identified simply by tBLASTn. This may have been adequate for the more closely related Drosophila PR genes, but identifying true orthologs can sometimes be a tricky business. It would help if you could provide a bit more detail in the methods about how you went about identifying and confirming orthologs.

More substantial but discretionary suggestions

* Just a thought, but would you consider that the Blimp1 RNAi phenotype suggests a gap function? Is Blimp1 a gap gene in Vanessa? If so, would this be worth a mention in the discussion?

* Although the pdf I was provided isn't the greatest quality, I think I can see that the seven or so stripes of Vcar-eve expression shown in Fig 3Civ are of alternating intensity, suggesting the existence of secondary segmental stripes, as found with Drosophila eve. This would constitute further evidence of an underlying PR mechanism and, if you agree, might be worth a mention in Lines 204-208.

* When you describe the PR-like knockdown phenotype for Vcar-eve, consider whether it might be worth it to mention in the main text the percentages you got for that phenotype (31% with dsRNA1 and 39% with dsRNA2, I think).

* When you describe the Vcar-Blimp1-dsRNA segmental fusion in 5Ei (Line 371 and Lines 806-807) can the fusion shown not be explicitly identified as A3 + A4? If so, consider adding that information.

© 2026. Published by The Company of Biologists under the terms of the Creative Commons Attribution License (https://creativecommons.org/licenses/by/4.0/).

* Lines 388-406: In this subsection of the discussion, it might be worth briefly reminding the reader of the Anopheles data, since I'm assuming that this is what motivated the investigation of gsb as a possible PR gene.

* Lines 408-423: Since you mention Vcar-odd in this subsection, would it also make sense to mention the data for Vcar-h?

* Line 475: You mention that you "classified opa expression as PR stripes", but is that correct? Do you mean segmental stripes?

Additional minor (also discretionary) suggestions

* All nouns modified by "PR" were hyphenated, as in "PR-stripes", "PR-genes" or "PR-expression", even when not used as compound adjectives; "simultaneous segmentation" was also hyphenated in Lines 45-46. I personally found this unnecessary and a bit distracting but feel this is a stylistic decision that should be up to the authors.

* Lines 107-116: Consider changing the description of development here to the present tense (Line 107: are observed, Line 108: blastoderm forms, Line 111: compresses, Line 112: invaginates, Line 113: resembles, Line 114: starts to elongate, Line 115-116: are distinguishable, Line 116: continues to elongate)

* Line 132: Since the sentence is not technically a question, I think you need a period after "function".

* Line 153: cut "presented" (since you already have "appeared")

* Line 234: Consider "Vcar-h expression was observed" >> "Vcar-h stripes were observed" since, if I'm not mistaken, I see stripes.

* Line 258 (+ others): I personally don't think you need to mention the dsRNA concentration in the main text (adequate to be in methods). This applies to other uses of "1.5 uM".

* Line 268-269: Consider integrating this information (percentages for dsRNA2) into the sentence above that describes the percentages for dsRNA1 (Line 260).

* Line 281-282: Consider "After Vcar-slp1-dsRNA1-injection, most embryos…" >> "After Vcar-slp1-dsRNA1-injection, while most embryos…"

* Line 319: Need a comma after "47%"

* Line 323: Did you intend "4Fii" instead of "4Fi"?

* Line 380: You mention that "All Vanessa PR gene orthologs are expressed from blastoderm stage through germband elongation, but Vcar-ftz-f1" but this is not true for E75, correct? You may have been referring to orthologs of just the Drosophila PR genes, but in any case, this should be clarified.

* Line 395: "…late embryos, however we…" >> "…late embryos; however, we…"

* Line 398: Consider simplifying "in other lepidopterans and other arthropods examined" to "in other lepidopterans and arthropods"

* Line 399: Consider adding an "and" to "…truncated abdominal segments, and in some cases…"

* Line 402: Consider "those" >> "some"

* Line 410: Cut "presents"

© 2026. Published by The Company of Biologists under the terms of the Creative Commons Attribution License (https://creativecommons.org/licenses/by/4.0/).

* Line 415: Consider present tense for "…Bombyx odd shows an expression pattern…"

* Lines 427-428: I recommend putting the genera names in parentheses to make this sentence clearer.

* Line 435: Comma needed after "…Kilchherr et al. 1986)"

* Line 448: Consider cutting "have examined several"

* Line 449: Comma needed after "Nasonia"

* Lines 455-457: These two sentences on slp seem sort of tacked on the end of this paragraph and sort of break the flow from talking about eve to talking about run.

* Line 462: Consider "In functional experiments we observed…" >> "Here we report…"

* Line 464: Consider adding a comma and "as" so that it reads "…head-like structures, as observed in Tribolium"

* Line 465: Consider starting a new paragraph with "Two other genes…"

* Lines 466-472: It's a bit odd that you don't mention the Vanessa results here for odd and h, no?

* Line 470: Consider "…but knockdown of Tribolium or Dermestes h failed to reveal roles in segmentation" >> …but no role in segmentation in either Tribolium or Dermestes"

* Line 473: "…(Benedyk et al. 1994), however opa…" >> "…(Benedyk et al. 1994); however, opa…"

* Line 479: Consider "neither the expression nor function of opa is PR"

* Lines 482-483: Consider cutting "It was observed" so that it reads "Nasonia E75a displays a PR expression pattern (Taylor and Dearden 2022), but…"

* Line 487: Consider "the function and expression pattern" >> "the expression and function"

* Line 499: Hyphen for "12-hour light/dark cycle"

* Line 500: Caps for Multiple Species Diet

* Line 506: Cut "annotation" so that reads "The annotated Vanessa genome"

* Figure 5C and 5Di: Would it help to add a "T3" label (or maybe A1 and A2 labels) to both the control and knockdown embryos to highlight where the aberration is located? (By the way, the embryo in 5Dii is a nice addition and convincing!)

* Figure 6: I found this figure very helpful and I wonder if it would help to put a thin black line around the squares that represent the work of this publication (and indicate so in the caption).

* The formatting for Table S1 in the pdf I had was messed up (probably happened when the pdf was generated), so just be sure to check on the tables.

Reviewer 2

Review of Ramos et al. Segmentation gene expression and function in _Vanessa cardui_, an emerging model for Lepidoptera

© 2026. Published by The Company of Biologists under the terms of the Creative Commons Attribution License (https://creativecommons.org/licenses/by/4.0/).

Thank you for the opportunity to review this manuscript. This is a well-written and well-performed piece of science. The authors investigate the role of pair-rule genes in the development of a lepidopteran, focussing on changes in the pair-rule gene network in this species, compared to others.

The experiments described are elegantly performed and include both gene expression studies, and functional studies, using RNA interference that test the function so the genes involved. This work clearly demonstrates that the pair-rule gene network of Vanessa differs from Drosophila, studied coleopterans and hymenoptera. The authors also test novel pair rule genes identified in hemimetabolous insects, and show they do not have a pair rule function in Vanessa. The manuscript provides very useful data, in an understudied group, about the evolution of insect segmentation mechanisms.

I believe this to be an excellent piece of work, which functionally tests the genes described, and is worthy of publication in Development.

I have a few issues that I think should be addressed before publication.

1) The differences between different forms of segmentation in insects have been well modelled by Clark and Peel, and Clark and Akam. It would be useful to discuss how this data tests and agrees with, or disagrees with this model.

2) It is a pity that the in-situ data hasn't used a fluorescent method that would have allowed for detection of multiple genes in each sample and perhaps giving hints about changes in the regulation network. Given the data presented, is it possible to discuss the potential changes in the regulation of genes in the segmentation cascade?

3) Can the authors increase the brightness of figures 2b, c and d, 4 an and b and 5c to improve clarity?

4) I think it vital to do careful phylogenetics of the proteins encoded by the genes studied here. The authors point out the complexity of paired and gooseberry, and there is always complexity of engrailed and invected. Solid phylogenetics will help assure that we are looking at the best orthologues of the genes studied. There is always the possibility of gene duplications or gene conversion that could muddy the identification of genes via blast searches alone.

<u>Reviewer 3</u>

SUMMARY OF THE ADVANCE MADE IN THIS PAPER AND ITS POTENTIAL SIGNIFICANCE TO THE FIELD

This manuscript provides an extensive description of the expression and function of pair-rule (PR) gene orthologs in Vanessa cardui, establishing it as another lepidopteran model for segmentation. 11 Drosophila PR-gene orthologs plus two Oncopeltus-specific PR genes (E75 and Blimp1), are described, using in situ hybridization and embryonic RNAi (eRNAi) knockdown. The work is of high quality and makes a valuable contribution to the field. They find that Vcar-gsb functions as a segment polarity gene rather than a PR gene; that Vcar-eve has classical PR function while Vcar-run likely does too; that Vcar-slp2 affects segmentation without classical PR output; and that Vcar-Blimp1 plays a novel, non-PR role in specifying only two abdominal segments.

SUGGESTIONS TO AUTHORS

The core findings are interesting and well-supported. The primary issues are: (1) inconsistency in how strongly Vcar-run function is claimed across sections of the paper; (2) insufficient mechanistic discussion of the Blimp1 phenotype; (3) the need for more careful framing of the "extensive rewiring" conclusion; and (4) several figure legend inaccuracies. Addressing these would strengthen the manuscript.

Major points:

© 2026. Published by The Company of Biologists under the terms of the Creative Commons Attribution License (https://creativecommons.org/licenses/by/4.0/).

1. Vcar-eve: The PR-like loss of even-numbered abdominal segments in Vcar-eve RNAi (Figure 4B) is the strongest result in the paper, and Figure 4B is convincing. However, this canonical phenotype represents only a subset of affected embryos (55% of dsRNA1-injected and 59% of dsRNA2-injected embryos) show segmentation defects in total, but these comprise a mixture of the PR-like class, head-like truncations, and partial fusions. A more explicit breakdown of what fraction of phenotypic embryos showed the clean PR-like class specifically would be important (Table S1 provides counts, but this is not discussed in the main text). The head-like and partial fusion phenotypes complicate the interpretation: are these simply more severe knockdowns, or do they reflect functions distinct from PR-patterning? A brief mechanistic discussion of how the PR-like, head-like, and fusion classes relate to one another (e.g., dose-dependence as shown for gsb) would strengthen the conclusions.

2. Vcar-run: The authors acknowledge in the Results that "we were unable to definitively determine whether Vcar-run has PR-like function" (lines 336-338) because the register of segmental deletions could not be resolved. However, the abstract states that "Vcar-run knockdown revealed PR-like function" and the Discussion treats it similarly. The text should be made internally consistent: either the evidence is sufficient to claim PR function (in which case the caveat in the Results needs revisiting) or it is not (in which case the abstract and Discussion claims need softening). Expression pattern alone is not equivalent to functional evidence.

3. Blimp1: Loss of specifically A3+A4 is a clean result (Figures 5D-E, high penetrance with both dsRNAs). However, the authors do not address why these segments are specifically vulnerable. expression at the relevant stages shows two widely spaced stripes anterior to the SAZ (Figure 5Biv), which the authors do not connect mechanistically to the identity of A3+A4. It makes sense that these are A3/4 but evidence is not shown. Also, does Blimp1 interact with any of the other genes characterized here? Are the epistatic relationships known? This result is highlighted in the abstract and conclusion as evidence of repeated evolutionary change in Blimp1 function, it deserves deeper treatment. Some discussion about what is known about Blimp1 molecular function and how that might explain a segment-specific role would be helpful.

4. Staging: Vanessa eggs are collected over an 8-hour window (Methods, lines 518-519). For some genes (e.g., Vcar-odd, Vcar-h), expression is transient and fades by 16 hours, raising the question of whether absence of expression in older germbands reflects genuine absence or simply that expression was earlier and was missed by the collection window. This matters for interpreting the Figure 6 comparative matrix, which codes genes as expressed or not in Vanessa. The authors should mention as a caveat how the 8-hour window might create gaps in detection.

5. extensive GRN rewiring: "Rewiring" implies that regulatory connections between genes have changed, not simply that the number of PR genes differs. The claim would be more defensible if phrased in terms of the PR-gene repertoire being reduced or divergent relative to beetles and other holometabolous insects.

Minor points:

Line 97-98: "Overall, Vanessa appears to utilize only a subset of the PR-gene orthologs found in other insects for PR-patterning." Which insects are the reference: the subset is relative to Drosophila but aligns more closely with some other holometabolous insects (discussed later). Clarify here.

Figure 3, Vcar-eve panels (C): The legend states "5 stripes along the germband" in panel (ii) and "4 stripes in the posterior" in panel (iii), yet the main text at line 205-206 says the posterior band split into "three separate stripes" at the stage corresponding to panel (ii). There is an inconsistency between the figure legend and main text stripe counts that should be reconciled.

Figure 4B, Vcar-eve knockdown: The identified segments (T2, A1, A3, A5, A7, A9 retained) match a classical PR-like pattern, but the legend does not state which even-numbered segments are absent. The PR interpretation depends entirely on which segments are missing, this should be stated explicitly: "A2, A4, A6, A8, A10 are absent."

© 2026. Published by The Company of Biologists under the terms of the Creative Commons Attribution License (https://creativecommons.org/licenses/by/4.0/).

Figure 6 comparative matrix: Several cells are colored for Vanessa expression without functional tests (e.g., Vcar-odd, Vcar-h, Vcar-opa, Vcar-ftz). The legend does not make sufficiently clear which functional data are from this paper versus inferred. The functional triangle for untested genes should be grey (no information), as the authors do for other species where data are unavailable.

Lines 447-448: "eve is the only gene with PR-expression and function across holometabolous insects examined to date." This is a strong statement and is one of the most important comparative conclusions of the paper. It deserves a dedicated discussion sentence. Why might eve be the most conserved PR gene? Its deep conservation is already known; the authors' contribution is adding Lepidoptera to that picture. This should be explicitly framed as confirmation and extension of a known principle.

Methods — embryonic RNAi: The rationale for using different concentrations should be explained. The injection concentration used for most genes is 1.5 μM, but Vcar-slp1 and Vcar-E75 were injected at 3 μM (Table S1). This difference is not discussed and may complicate cross-gene comparisons, particularly since the negative result for Vcar-slp1 at high concentration differs from the positive result for Vcar-slp2 at 1.5 μM.

Statistical reporting: Phenotype frequencies are reported throughout but no formal statistical comparisons between dsRNA1 and dsRNA2 penetrance are presented in the main text. Given that the two dsRNAs sometimes give substantially different penetrance (e.g., Blimp1: 79% vs. 56%), readers need some basis for judging whether these differences are meaningful. A chi-square or Fisher's exact test comparison of dsRNA1 vs. dsRNA2 phenotype frequencies, noted in the text or table, would be appropriate.

Minor issues in text Line 22: "Blimp1 functions in a novel fashion" —"non-PR fashion" would be more precise and appropriate.

Line 98: "a more limited or divergent set of genes" — choose one; these have different implications.

First revision

<u>Author response to reviewers' comments</u>

We thank the reviewers for the largely positive reviews of our manuscript and for noting areas in need of correction or clarification. Large changes include: a new Table S6 and Figure S2 to confirm orthology of the *Vanessa* genes we isolated; improvements to the contrast and brightness in Figures 2B, 2C, 2D, 4A, 4B, 5C; providing a different example for Figure 3 Fiii to more clearly show the expression pattern described; and a new Table S2 with statistical analysis. Below we detail our response to each reviewer comment in blue. The line numbers refer to the marked-up version.

Reviewer 1: SUMMARY OF THE ADVANCE MADE IN THIS PAPER AND ITS POTENTIAL SIGNIFICANCE TO THE FIELD

The paper takes a candidate gene approach to examine potential pair-rule (PR) expression and function in the butterfly, Vanessa cardui. The work is novel in investigating a butterfly (previous lepidopteran studies have focused on moths) and aside from documenting expression patterns of eleven orthologs of PR genes known from Drosophila and Oncopeltus (and the RNAi phenotypes of six), primary contributions include 1) a convincing demonstration that V. cardui even-skipped (Vcar-eve) is both expressed and functions as a pair-rule gene (Fig. 4B) and 2) the discovery that V. cardui Blimp1 plays a novel role in abdominal segmentation (Fig. 5D). These discoveries contribute to our overall picture of how the genetic and developmental mechanisms underlying a conserved morphology (in this case, segmentation) evolve.

SUGGESTIONS TO AUTHORS

© 2026. Published by The Company of Biologists under the terms of the Creative Commons Attribution License (https://creativecommons.org/licenses/by/4.0/).

While I found the evidence for PR expression and function for Vcar-eve (Figs. 3C and 4B) compelling—very cool how the lepidopteran prolegs helped here—I was less convinced by the evidence presented for V. cardui runt (Vcar-run) (Figs. 3F and 4F). I agree that the evidence is consistent with a possible PR role, but it did seem to fall short of being definitive on this point. As an easy remedy, here are several points in the introduction and discussion where the language could be qualified to bring it more in line with the stated results (e.g., Lines 324-325 and 337-338):

Yes, we agree that the *run* status is a bit ambiguous (as it is in *Oncopeltus* as well) and that the presentation should be corrected. We appreciate the reviewer pointing this out. We have made changes throughout the text accordingly:

* Line 94: The use of "or" seems odd in this sentence. As expressed above, I also have some reservations about the PR claims for Vcar-run. Thus, consider: "…RNAi knockdown of Vcar-eve, and possibly Vcar-run, revealed PR-like function."

Changed as suggested. Line 97

* Line 223: Here there is mention of Vcar-run "stripes" in the posterior of the germband and in the SAZ. It may be the quality of the pdf I received, but I'm having a hard time seeing these stripes in Fig 3Fiii. If anything, I see broad expression throughout the SAZ, and even that's not entirely clear. Consider revisiting this description.

The description is correct; we observed stripes across the posterior part of the germband. You are correct that the stripes were hard to observe in Fig3 Fiii, so we have changed the photo in this panel to one that better shows the striped expression pattern in the SAZ.

* Lines 382-383: Consider changing "…classic PR-function for Vcar-eve while revealed Vcar-run knockdown embryos displayed fusion or loss of some or all segments, likely indicative of PR-function." >> "…classic PR function for Vcar-eve, and a segmental role, possibly PR, for Vcar-run."

Changed to now read "and classic PR-function for *Vcar-eve*. *Vcar-run* knockdown embryos displayed fusion or loss of some or all segments, possibly indicative of PR-function" Lines 387-388

* Line 414: Add "possibly" so that it reads "…and possibly run appear…"

Since this refers to expression pattern, we did not add "possibly" but include "appear" in this sentence. Lines 416-417

* Figure 6: Add a "?" to the PR function wedge in the square for Vcar-run to indicate some remaining uncertainty.

We added the "?" to indicate the uncertainty of the result. Updated in Figure 6.

* Line 462: Change "likely" to "possible"

This section was revised to in accordance with reviewer suggestions and now reads "Similarly, PR-expression for *run* is shared by many holometabolous insects, having been documented for *Drosophila, Bombyx, Manduca, Tribolium, Dermestes, Nasonia, Apis* (Gergen and Butler 1988; Kraft and Jackle 1994; Choe et al. 2006; Liu et al. 2008; Wilson and Dearden 2012; Rosenberg et al. 2014; Nakao 2015; Xiang et al. 2017; Taylor and Dearden 2022). In the hemipteran insect *Oncopeltus*, *run* was the only *Drosophila* PRG-ortholog with an expression pattern resembling PR, although RNAi failed to reveal PR-function (Reding 2019). Our functional experiments in *Vanessa* revealed a possible PR-like of loss of alternate segments, as well as a severe knockdown phenotype with only head-like structures, similar to *run* knockdown phenotypes in *Tribolium*, *Dermestes* and *Bombyx*". Lines 465-474

I realize that the difference between "likely" and "possible" is small and if you prefer the former, I suppose I would request that you explain why you believe a PR role is likely versus simply possible based on the evidence presented.

© 2026. Published by The Company of Biologists under the terms of the Creative Commons Attribution License (https://creativecommons.org/licenses/by/4.0/).

Agreed and we made the requested changes. It is because of its expression pattern and the truncated phenotypes we see for *Vcar-run* that we used the original wording. The truncated phenotypes are typical of pair-rule gene knockdown in sequentially-segmenting species. However, we agree that without stronger evidence, it is best to use more cautious wording. See current lines 341-344.

As described in the methods, Vcar orthologs were identified simply by tBLASTn. This may have been adequate for the more closely related Drosophila PR genes, but identifying true orthologs can sometimes be a tricky business. It would help if you could provide a bit more detail in the methods about how you went about identifying and confirming orthologs.

To complement the section on the identification of *Vanessa* orthologs of segmentation genes we have added a phylogenetic analysis. This new information is reported in Table S6 and Figure S2 and explained in Methods:

"Once *Vanessa* segmentation genes were identified, each protein domain was manually annotated, indicated in Figure S1. Next, we selected and identified segmentation protein sequences from different arthropods (see Table S6) to perform phylogenetic reconstructions (Fig. S2). Sequence alignment used MAFFT v.7.511 (Katoh and Standley 2013) with L-INS-i model and arguments: –phylipout –reorder. The maximum likelihood trees were built with IQ-Tree 3.1.2 (Wong et al. 2026), using the model Q.insect+I+R4, with 1,000 ultrafast bootstraps (Hoang et al. 2018). Visualizations were carried out using ggtree v.3.23 (Yu et al. 2017) and Inkscape v.1.3.2." Lines 197; 517-526.

<u>More substantial but discretionary suggestions</u>

* Just a thought, but would you consider that the Blimp1 RNAi phenotype suggests a gap function? Is Blimp1 a gap gene in Vanessa? If so, would this be worth a mention in the discussion?

Yes, we agree. By the "classic" *Drosophila*-centric groupings of genes, it would be gap-like but overall work in sequentially segmenting species suggests that these categories may not neatly apply (e.g., *eve*, *odd* and *run* knockdowns in sequentially segmenting species result in head-only phenotypes, likely reflecting a role of PRGs that is not required/utilized in *Drosophila*). This is probably better addressed in a review article than in this particular research article. Thus, we added this to the Discussion "Knockdown of *Vcar-Blimp1* produced fusions of abdominal segments A3 and A4 (Figs. 2, 4, S3, 5), possibly indicative of gap-like function although gap-like expression was not observed." Lines 389-390

* Although the pdf I was provided isn't the greatest quality, I think I can see that the seven or so stripes of Vcar-eve expression shown in Fig 3Civ are of alternating intensity, suggesting the existence of secondary segmental stripes, as found with Drosophila eve. This would constitute further evidence of an underlying PR mechanism and, if you agree, might be worth a mention in Lines 204-208.

We don't feel confident that this is the case.

* When you describe the PR-like knockdown phenotype for Vcar-eve, consider whether it might be worth it to mention in the main text the percentages you got for that phenotype (31% with dsRNA1 and 39% with dsRNA2, I think).

We added the percentages for the *Vcar-eve* dsRNA main phenotypes. Spanning lines 257-266

* When you describe the Vcar-Blimp1-dsRNA segmental fusion in 5Ei (Line 371 and Lines 806-807) can the fusion shown not be explicitly identified as A3 + A4? If so, consider adding that information.

We changed "segmental fusions were observed (Fig. 5Ei, arrowheads)" to "fusions of segments A3+A4" Line 376

* Lines 388-406: In this subsection of the discussion, it might be worth briefly reminding the reader of the Anopheles data, since I'm assuming that this is what motivated the investigation of gsb as a possible PR gene.

© 2026. Published by The Company of Biologists under the terms of the Creative Commons Attribution License (https://creativecommons.org/licenses/by/4.0/).

We added “Because *gsb* has replaced *prd* in mosquitoes (Cheatle Jarvela et al. 2020), we investigated *gsb* expression and function in more detail in Lepidoptera.” Current line 397-399.

* Lines 408-423: Since you mention Vcar-odd in this subsection, would it also make sense to mention the data for Vcar-h?

In this section, we only mention PR-data for genes examined in other lepidopteran species. We are not aware of any information for expression or function of *Bombyx*, *Manduca* or *Bicyclus hairy*.

* Line 475: You mention that you "classified opa expression as PR stripes", but is that correct? Do you mean segmental stripes?

Thank you for catching this error. Now reads “In *Vanessa*, *opa* is expressed in segmental stripes,” Lines 484-485

<u>Additional minor (also discretionary) suggestions</u>

* All nouns modified by "PR" were hyphenated, as in "PR-stripes", "PR-genes" or "PR-expression", even when not used as compound adjectives; "simultaneous segmentation" was also hyphenated in Lines 45-46. I personally found this unnecessary and a bit distracting but feel this is a stylistic decision that should be up to the authors.

Quite honestly, this helps us with the limit on number of words allowed.

* Lines 107-116: Consider changing the description of development here to the present tense (Line 107: are observed, Line 108: blastoderm forms, Line 111: compresses, Line 112: invaginates, Line 113: resembles, Line 114: starts to elongate, Line 115-116: are distinguishable, Line 116: continues to elongate)

I realize that there are different philosophies about this, but I prefer to put in the past tense any result we observed under a particular condition. For example, the time of blastoderm formation may differ depending on environmental conditions. My philosophy is that if we say what we did and what we saw, we cannot be wrong. If we say “8 hour AEL is when blastoderm forms” as a universal, the statement will likely not be correct.

* Line 132: Since the sentence is not technically a question, I think you need a period after "function".

True, corrected, thank you. Line 134

* Line 153: cut "presented" (since you already have "appeared"). Done. Line 155

* Line 234: Consider "Vcar-h expression was observed" >> "Vcar-h stripes were observed" since, if I'm not mistaken, I see stripes.

We left this sentence as is but added *Vcar-h* to the start of the following sentence. Line 236

* Line 258 (+ others): I personally don't think you need to mention the dsRNA concentration in the main text (adequate to be in methods). This applies to other uses of "1.5 uM".

We removed the dsRNA concentration from Results, Lines 256 and 322

* Line 268-269: Consider integrating this information (percentages for dsRNA2) into the sentence above that describes the percentages for dsRNA1 (Line 260).

Done. Changes between lines 258 and 270

* Line 281-282: Consider "After Vcar-slp1-dsRNA1-injection, most embryos…" >> "After Vcar-slp1-dsRNA1-injection, while most embryos…"

© 2026. Published by The Company of Biologists under the terms of the Creative Commons Attribution License (https://creativecommons.org/licenses/by/4.0/).

Added “while” Line 288

* Line 319: Need a comma after "47%".

Done. Line 325

* Line 323: Did you intend "4Fii" instead of "4Fi"?

Yes, corrected, thank you. Line 328

* Line 380: You mention that "All Vanessa PR gene orthologs are expressed from blastoderm stage through germband elongation, but Vcar-ftz-f1" but this is not true for E75, correct? You may have been referring to orthologs of just the Drosophila PR genes, but in any case, this should be clarified.

Correct. We changed the wording to clarify “Except *Vcar-ftz-f1*, the orthologs of all *Drosophila* PR-genes were expressed from blastoderm stage through germband elongation in *Vanessa*” Lines 385-386

* Line 395: "...late embryos, however we..." >> "...late embryos; however, we...".

Done. Line 402

* Line 398: Consider simplifying "in other lepidopterans and other arthropods examined" to "in other lepidopterans and arthropods".

Changed to “in other lepidopterans” Lines 404-406

* Line 399: Consider adding an "and" to "...truncated abdominal segments, and in some cases..."

Done. Line 407

* Line 402: Consider "those" >> "some"

Done. Line 410

* Line 410: Cut "presents". Done. Line 417

* Line 415: Consider present tense for "...Bombyx odd shows an expression pattern..."

As per my philosophy mentioned above, I prefer to leave it in the past tense.

* Lines 427-428: I recommend putting the genera names in parentheses to make this sentence clearer.

Done. Lines 434-435

* Line 435: Comma needed after "...Kilchherr et al. 1986)".

Done. Line 442

* Line 448: Consider cutting "have examined several".

Done. Line 455

* Line 449: Comma needed after "Nasonia".

Done. Line 456

© 2026. Published by The Company of Biologists under the terms of the Creative Commons Attribution License (https://creativecommons.org/licenses/by/4.0/).

* Lines 455-457: These two sentences on slp seem sort of tacked on the end of this paragraph and sort of break the flow from talking about eve to talking about run.

We see your point but we are discussing Fig. 6 in the order in which the genes are listed.

* Line 462: Consider "In functional experiments we observed..." >> "Here we report...".

Because of wording changes based on a comment above, “Here we report” no longer fits the sentence structure. Line 470-471

* Line 464: Consider adding a comma and "as" so that it reads "...head-like structures, as observed in Tribolium".

We changed some text in this paragraph as suggested above, so this change is no longer doable. This part now reads “severe knockdown phenotype with only head-like structures, similar to *run* knockdown phenotypes in *Tribolium*,” current lines 472-473

* Line 465: Consider starting a new paragraph with "Two other genes...".

Done. Line 475

* Lines 466-472: It's a bit odd that you don't mention the Vanessa results here for odd and h, no?

Just trying to keep the text shorter and less repetitive and we are unaware of any data for *hairy* or *odd* in other lepidopeteran insects.

* Line 470: Consider "...but knockdown of Tribolium or Dermestes h failed to reveal roles in segmentation" >> ...but no role in segmentation in either Tribolium or Dermestes".

Corrected to “but not in either *Tribolium* or *Dermestes”* Current lines 479-481

* Line 473: "...(Benedyk et al. 1994), however opa..." >> "...(Benedyk et al. 1994); however, opa...". Done. Line 483

* Line 479: Consider "neither the expression nor function of opa is PR" Done.Line 498

* Lines 482-483: Consider cutting "It was observed" so that it reads "Nasonia E75a displays a PR expression pattern (Taylor and Dearden 2022), but..."".

Done. Line 492-493

* Line 487: Consider "the function and expression pattern" >> "the expression and function". Done. Line 497

* Line 499: Hyphen for "12-hour light/dark cycle". Done. Line 510

* Line 500: Caps for Multiple Species Diet. Done. Line 510-511

* Line 506: Cut "annotation" so that reads "The annotated Vanessa genome".

Done. Line 517

* Figure 5C and 5Di: Would it help to add a "T3" label (or maybe A1 and A2 labels) to both the control and knockdown embryos to highlight where the aberration is located? (By the way, the embryo in 5Dii is a nice addition and convincing!).

We added A1 and A2 labels to Figure 5C and 5Di.

* Figure 6: I found this figure very helpful and I wonder if it would help to put a thin black line around the squares that represent the work of this publication (and indicate so in the caption).

© 2026. Published by The Company of Biologists under the terms of the Creative Commons Attribution License (https://creativecommons.org/licenses/by/4.0/).

Figure 6 We added the thin black line to Figure 6 and figure legend. Line 851,854

* The formatting for Table S1 in the pdf I had was messed up (probably happened when the pdf was generated), so just be sure to check on the tables.

Thank you, we will upload supplementary data as excel files and check the Tables after the PDF conversion.

Reviewer 2: Review of Ramos et al. Segmentation gene expression and function in _Vanessa cardui, an emerging model for Lepidoptera

Thank you for the opportunity to review this manuscript. This is a well-written and well-performed piece of science. The authors investigate the role of pair-rule genes in the development of a lepidopteran, focussing on changes in the pair-rule gene network in this species, compared to others.

The experiments described are elegantly performed and include both gene expression studies, and functional studies, using RNA interference that test the function so the genes involved. This work clearly demonstrates that the pair-rule gene network of Vanessa differs from Drosophila, studied coleopterans and hymenoptera. The authors also test novel pair rule genes identified in hemimetabolous insects, and show they do not have a pair rule function in Vanessa. The manuscript provides very useful data, in an understudied group, about the evolution of insect segmentation mechanisms.

I believe this to be an excellent piece of work, which functionally tests the genes described, and is worthy of publication in Development.

I have a few issues that I think should be addressed before publication.

1) The differences between different forms of segmentation in insects have been well modelled by Clark and Peel, and Clark and Akam. It would be useful to discuss how this data tests and agrees with, or disagrees with this model.

We are definitely aware of the importance of Erik Clark’s work and try to cite it regularly. However, we don’t see that our results contribute one way or the other to their analysis.

2) It is a pity that the in-situ data hasn't used a fluorescent method that would have allowed for detection of multiple genes in each sample and perhaps giving hints about changes in the regulation network. Given the data presented, is it possible to discuss the potential changes in the regulation of genes in the segmentation cascade?

This is an excellent goal for future experiments but is beyond the scope of the current study.

3) Can the authors increase the brightness of figures 2b, c and d, 4 an and b and 5c to improve clarity?

Yes, thank you for the suggestion. We increased the brightness and changed the color of the letters to improve contrast with the cuticle preps images. See updated Figures 2B, 2C, 2D, 4A, 4B and 5C.

4) I think it vital to do careful phylogenetics of the proteins encoded by the genes studied here. The authors point out the complexity of paired and gooseberry, and there is always complexity of engrailed and invected. Solid phylogenetics will help assure that we are looking at the best orthologues of the genes studied. There is always the possibility of gene duplications or gene conversion that could muddy the identification of genes via blast searches alone.

Thank you for your suggestion, which was also made by reviewer #1. As we responded above, we have now done and included a phylogenetic analysis of each gene. The information that we obtained is reported in Table S6 and Figure S2 and explained in Methods:

© 2026. Published by The Company of Biologists under the terms of the Creative Commons Attribution License (https://creativecommons.org/licenses/by/4.0/).

"Once *Vanessa* segmentation genes were identified, each protein domain was manually annotated, indicated in Figure S1. Next, we selected and identified segmentation protein sequences from different arthropods (see Table S6) to perform phylogenetic reconstructions (Fig. S2). Sequence alignment used MAFFT v.7.511 (Katoh and Standley 2013) with L-INS-i model and arguments: –phylipout –reorder. The maximum likelihood trees were built with IQ-Tree 3.1.2 (Wong et al. 2026), using the model Q.insect+I+R4, with 1,000 ultrafast bootstraps (Hoang et al. 2018). Visualizations were carried out using ggtree v.3.23 (Yu et al. 2017) and Inkscape v.1.3.2.". Lines 197; 517-526.

Reviewer 3: SUMMARY OF THE ADVANCE MADE IN THIS PAPER AND ITS POTENTIAL SIGNIFICANCE TO THE FIELD

This manuscript provides an extensive description of the expression and function of pair-rule (PR) gene orthologs in Vanessa cardui, establishing it as another lepidopteran model for segmentation. 11 Drosophila PR-gene orthologs plus two Oncopeltus-specific PR genes (E75 and Blimp1), are described, using in situ hybridization and embryonic RNAi (eRNAi) knockdown. The work is of high quality and makes a valuable contribution to the field. They find that Vcar-gsb functions as a segment polarity gene rather than a PR gene; that Vcar-eve has classical PR function while Vcar-run likely does too; that Vcar-slp2 affects segmentation without classical PR output; and that Vcar-Blimp1 plays a novel, non-PR role in specifying only two abdominal segments.

SUGGESTIONS TO AUTHORS

The core findings are interesting and well-supported. The primary issues are: (1) inconsistency in how strongly Vcar-run function is claimed across sections of the paper; (2) insufficient mechanistic discussion of the Blimp1 phenotype; (3) the need for more careful framing of the "extensive rewiring" conclusion; and (4) several figure legend inaccuracies. Addressing these would strengthen the manuscript.

Major points:

1. Vcar-eve: The PR-like loss of even-numbered abdominal segments in Vcar-eve RNAi (Figure 4B) is the strongest result in the paper, and Figure 4B is convincing. However, this canonical phenotype represents only a subset of affected embryos (55% of dsRNA1-injected and 59% of dsRNA2-injected embryos) show segmentation defects in total, but these comprise a mixture of the PR-like class, head-like truncations, and partial fusions. A more explicit breakdown of what fraction of phenotypic embryos showed the clean PR-like class specifically would be important (Table S1 provides counts, but this is not discussed in the main text). The head-like and partial fusion phenotypes complicate the interpretation: are these simply more severe knockdowns, or do they reflect functions distinct from PR-patterning? A brief mechanistic discussion of how the PR-like, head-like, and fusion classes relate to one another (e.g., dose-dependence as shown for gsb) would strengthen the conclusions.

These types of findings are typical for RNAi experiments, and the specific phenotypes are expected for pair-rule genes in sequentially segmenting species. We changed this section of the Results to "The range of phenotypes observed is typical for RNAi knockdown, which generates the equivalent of an allelic series due to differences in dose or timing of dsRNA action in different embryos. For beetles, knockdown of *eve* generated a similar range of defects with strong knockdown resulting in head-only phenotypes and weak knockdown producing partial segmental fusions with PR-register (Choe et al. 2006; Xiang et al. 2017). The truncations seen for eve and other PR-genes in sequentially-segmenting species likely reflect a role not required in simultaneously segmenting specific like *Drosophila*. In sum, these results suggest that *Vcar-eve* has PR-function in *Vanessa*." Lines 270-277

2. Vcar-run: The authors acknowledge in the Results that "we were unable to definitively determine whether Vcar-run has PR-like function" (lines 336-338) because the register of segmental deletions could not be resolved. However, the abstract states that "Vcar-run knockdown revealed PR-like function" and the Discussion treats it similarly. The text should be made internally consistent: either the evidence is sufficient to claim PR function (in which case the caveat in the Results needs revisiting) or it is not (in which case the abstract and Discussion claims need softening). Expression pattern alone is not equivalent to functional evidence.

© 2026. Published by The Company of Biologists under the terms of the Creative Commons Attribution License (https://creativecommons.org/licenses/by/4.0/).

Yes, reviewer #1 made this point as well and we agree. We made changes throughout regarding this conclusion about *runt* and listed those as responses to the specific edit requests of reviewer #1 above. For example, at the end of the Introduction, we changed the sentence with *runt* to read "Orthologs of four PR-genes have PR-like expression in *Vanessa* and RNAi knockdown of *Vcar-eve*, and possibly *Vcar-run*, revealed a PR-like function." Lines 96-97. There is no mention of *runt* in the Abstract.

3. Blimp1: Loss of specifically A3+A4 is a clean result (Figures 5D-E, high penetrance with both dsRNAs). However, the authors do not address why these segments are specifically vulnerable. expression at the relevant stages shows two widely spaced stripes anterior to the SAZ (Figure 5Biv), which the authors do not connect mechanistically to the identity of A3+A4. It makes sense that these are A3/4 but evidence is not shown. Also, does Blimp1 interact with any of the other genes characterized here? Are the epistatic relationships known? This result is highlighted in the abstract and conclusion as evidence of repeated evolutionary change in Blimp1 function, it deserves deeper treatment. Some discussion about what is known about Blimp1 molecular function and how that might explain a segment-specific role would be helpful.

Thank you for this comment. We agree that the *Blimp1* expression pattern does not point to a specific role in A3/A4. That is, we have not been able to reconcile the Vcar-Blimp1 expression pattern with its knockdown phenotype. We have added to line 380 "Knockdown of *Vcar-Blimp1* produced fusions of abdominal segments A3 and A4 (Figs. 2, 4, S3, 5), possibly indicative of gap-like function, although gap-like expression was not observed." Line 390

Unfortunately, the only information we have about the role of *Blimp1* in segmentation comes from our own 2024 paper which demonstrated its role as a pair-rule gene in *Oncopeltus*. For that species, we have not yet resolved regulatory or epistatic relationships. Thus we do not have a mechanistic explanation at this stage and are reporting the finding as such. Blimp1 functions as a transcription factor in many other pathways, including human immunity and a review article discussing its seemingly unusual versatility might be worthwhile. Given that we are already above the word limit for publication in Development, we are hesitant to add more text that would be purely speculative at this point and prefer to conclude with the sentence on lines 485-486 "How *Blimp1* fits into a segmentation gene network in butterflies remains to be investigated." Line 495-496

4. Staging: Vanessa eggs are collected over an 8-hour window (Methods, lines 518-519). For some genes (e.g., Vcar-odd, Vcar-h), expression is transient and fades by 16 hours, raising the question of whether absence of expression in older germbands reflects genuine absence or simply that expression was earlier and was missed by the collection window. This matters for interpreting the Figure 6 comparative matrix, which codes genes as expressed or not in Vanessa. The authors should mention as a caveat how the 8-hour window might create gaps in detection.

We agree that it is impossible to rule out having missed windows of transient expression but each collection and in situ contained multiple embryos at many stages. We added the caveat to Figure legend 6 "We note that we cannot rule out, for our studies and likely for published work of others as well, short periods of transient expression missed due to embryo staging." Lines 859-860

5. extensive GRN rewiring: "Rewiring" implies that regulatory connections between genes have changed, not simply that the number of PR genes differs. The claim would be more defensible if phrased in terms of the PR-gene repertoire being reduced or divergent relative to beetles and other holometabolous insects.

This is a very fair point. We have removed this sentence from the Abstract and will address regulatory interactions in future studies. We replaced this sentence with "How *Vanessa* patterns segments with a more limited set of PR-gene orthologs remains to be determined." Lines 25-27

Minor points:

Line 97-98: "Overall, Vanessa appears to utilize only a subset of the PR-gene orthologs found in other insects for PR-patterning." Which insects are the reference: the subset is relative to

© 2026. Published by The Company of Biologists under the terms of the Creative Commons Attribution License (https://creativecommons.org/licenses/by/4.0/).

Drosophila but aligns more closely with some other holometabolous insects (discussed later). Clarify here.

We changed this to “Overall, *Vanessa* appears to utilize a subset of the *Drosophila* PR-genes, but none of the *Oncopeltus* orthologs, for PR-patterning.” Lines 100-102

Figure 3, Vcar-eve panels (C): The legend states "5 stripes along the germband" in panel (ii) and "4 stripes in the posterior" in panel (iii), yet the main text at line 205-206 says the posterior band split into "three separate stripes" at the stage corresponding to panel (ii). There is an inconsistency between the figure legend and main text stripe counts that should be reconciled.

This wording has been clarified with: “*Vcar-eve* expression was first detected in the blastoderm as 2 anterior stripes and a wide band in the posterior (Fig. 3Ci, arrowheads). Later, this posterior band appeared to split into three separate stripes (Fig. 3Cii), resulting in five bands across the germband primordia”. Lines 206-208

Figure 4B, Vcar-eve knockdown: The identified segments (T2, A1, A3, A5, A7, A9 retained) match a classical PR-like pattern, but the legend does not state which even-numbered segments are absent. The PR interpretation depends entirely on which segments are missing, this should be stated explicitly: "A2, A4, A6, A8, A10 were lost."

Done. Lines 823-825

Figure 6 comparative matrix: Several cells are colored for Vanessa expression without functional tests (e.g., Vcar-odd, Vcar-h, Vcar-opa, Vcar-ftz). The legend does not make sufficiently clear which functional data are from this paper versus inferred. The functional triangle for untested genes should be grey (no information), as the authors do for other species where data are unavailable.

We clarified the Figure 6 Legend, with "the expression pattern *and/or* functional", Line 851. We added to Figure 6 an asterisk and rectangle to denote the information that we presented in this paper (*Vcar* column) and added "(present work, asterisk and thin black line) " to the legend. Line 854

Lines 447-448: "eve is the only gene with PR-expression and function across holometabolous insects examined to date." This is a strong statement and is one of the most important comparative conclusions of the paper. It deserves a dedicated discussion sentence. Why might eve be the most conserved PR gene? Its deep conservation is already known; the authors' contribution is adding Lepidoptera to that picture. This should be explicitly framed as confirmation and extension of a known principle

We suspect this may be simply historical - more studies looked at *eve* in other insects than at other pair-rule genes. Therefore, this may not reflect a uniquely conserved role for *eve*. We state, “although more studies have examined *eve* than other PR-gene orthologs”. Line 455-456

Methods — embryonic RNAi: The rationale for using different concentrations should be explained. The injection concentration used for most genes is 1.5 μM, but Vcar-slp1 and Vcar-E75 were injected at 3 μM (Table S1). This difference is not discussed and may complicate cross-gene comparisons, particularly since the negative result for Vcar-slp1 at high concentration differs from the positive result for Vcar-slp2 at 1.5 μM.

We added a clarification on the use of different concentrations of dsRNAs on Methods, “For all eRNAi injections (Table 8), 1.5 μM dsRNA of each dsRNA was injected first. For most of the genes, we observed a segmentation defect phenotype using this concentration. For *Vcar-slp1* and *Vcar-E75,* we did not observe defects so we increased the concentration to 3 μM to rule out the possibility that the dose of dsRNA was too low. However, we still observed wildtype-like phenotypes (Table S1).” Lines 570-574

Statistical reporting: Phenotype frequencies are reported throughout but no formal statistical comparisons between dsRNA1 and dsRNA2 penetrance are presented in the main text. Given that

© 2026. Published by The Company of Biologists under the terms of the Creative Commons Attribution License (https://creativecommons.org/licenses/by/4.0/).

the two dsRNAs sometimes give substantially different penetrance (e.g., Blimp1: 79% vs. 56%), readers need some basis for judging whether these differences are meaningful. A chi-square or Fisher's exact test comparison of dsRNA1 vs. dsRNA2 phenotype frequencies, noted in the text or table, would be appropriate.

We have completed a chi-square statistical analysis and include that in new Table S2. This showed that both dsRNAs for all the genes that we tested are non-independent, meaning that percentages from both dsRNAs have a statistically significant association. However, our understanding of the RNAi field is that variation in the effectiveness of different dsRNAs targeting the same gene is very common. This may be because of sequence composition, secondary structure in the target RNA, differential accessibility of different regions of the target RNA for base pairing, the generation of secondary RNAs, or more. To our knowledge, it has not been possible to derive the principles that would allow for efficiency predictions for RNAi in any system. Therefore, our use of two different dsRNAs is more qualitative, to ensure ourselves that we are not scoring a phenotype that is caused by off-target matches to a different mRNA. As long as we don't see different phenotypes for the two non-overlapping dsRNAs, we are able to conclude that the phenotypes we report are a result of knockdown of the gene targeted. We have no expectation that penetrance will be the same for the two dsRNAs. Differences are likely meaningful in terms of RNAi mechanism of action but not in terms of the function of the targeted mRNA.

Text addition: "Briefly, for each gene knockdown, we used two non-overlapping dsRNAs, which were injected and then observed phenotypes were scored (Table S1). For each gene, a chi-square statistical test was performed to compare dsRNA1 vs. dsRNA2 (Table S2), which showed that for all genes, both dsRNAs are non-independent, and therefore both dsRNAs have a statistically significant association." Lines 566-570

Minor issues in text

Line 22: "Blimp1 functions in a novel fashion" —"non-PR fashion" would be more precise and appropriate.

To our knowledge, the A3/A4 fusion is indeed a novel phenotype, and Blimp1 has not been shown to play this role in any other species. I know we over-hype with the use of "novel" but I think it is appropriate in this case.

Line 98: "a more limited or divergent set of genes" — choose one; these have different implications.

We changed this to clarify that we meant the two different possibilities "a more limited or as yet undiscovered." With the "or" we acknowledge that these are different scenarios, and we do not yet know which one applies – *Vanessa* use fewer genes or they use different genes. Lines 102-103

Second decision letter

MS ID#: dev.205835R1

MS TITLE: Segmentation gene expression and function in Vanessa cardui, an emerging model for Lepidoptera

AUTHORS: Ximena Gutiérrez Ramos; Katie Reding; Leslie Pick

Dear Dr Pick,

I am happy to tell you that your manuscript has been accepted for publication in Development, pending our standard publication integrity checks.

© 2026. Published by The Company of Biologists under the terms of the Creative Commons Attribution License (https://creativecommons.org/licenses/by/4.0/).

Reviewer 1

The authors have done a thorough job of responding to feedback, have accepted almost all my suggestions, and had good rationales for when they did not. I was particularly happy to see the appropriate caveats added to the main text, as well as a phylogenetic analysis for assigning orthology, which was added as supplementary material (Fig. S2). I also appreciate the different image/embryo for the expression of Vcar-run (Fig. 3Fiii), which, I agree, makes a stronger case for stripes as opposed to broad expression.

I did note that in part of the rewritten text (line 468), there is perhaps an extra “of” so that it reads “revealed a possible PR-like of loss of alternate segments” when I think the intended language is “revealed a possible PR-like loss of alternate segments”.

Finally, the authors might consider bolding the Vcar genes in the trees shown in Fig. S2 to make it easier for readers to find them, but this is just a suggestion.

Reviewer 2

The authors have done a good job revising the manuscript in response to reviewers' comments. I recommend its publication.

Reviewer 3

SUMMARY OF THE ADVANCE MADE IN THIS PAPER AND ITS POTENTIAL SIGNIFICANCE TO THE FIELD

The manuscript describes the expression and function of pair-rule genes in an understudied insect model, and contributes to our understanding of PR gene evolution.

SUGGESTIONS TO AUTHORS

The authors have thoughtfully responded to the comments of all 3 Reviewers, and consequenlly made substantial improvements to the manuscript.

© 2026. Published by The Company of Biologists under the terms of the Creative Commons Attribution License (https://creativecommons.org/licenses/by/4.0/).
